# Diversity and Activity of Diazotrophs in Great Barrier Reef Surface Waters

**DOI:** 10.3389/fmicb.2017.00967

**Published:** 2017-06-07

**Authors:** Lauren F. Messer, Mark V. Brown, Miles J. Furnas, Richard L. Carney, A. D. McKinnon, Justin R. Seymour

**Affiliations:** ^1^Climate Change Cluster, School of Life Sciences, University of Technology Sydney, SydneyNSW, Australia; ^2^School of Biotechnology and Biomolecular Sciences, University of New South Wales, SydneyNSW, Australia; ^3^Australian Institute of Marine Science, TownsvilleQLD, Australia

**Keywords:** N_2_ fixation, Great Barrier Reef, diazotrophs, diversity, nifH amplicon sequencing

## Abstract

Discrepancies between bioavailable nitrogen (N) concentrations and phytoplankton growth rates in the oligotrophic waters of the Great Barrier Reef (GBR) suggest that undetermined N sources must play a significant role in supporting primary productivity. One such source could be biological dinitrogen (N_2_) fixation through the activity of “diazotrophic” bacterioplankton. Here, we investigated N_2_ fixation and diazotroph community composition over 10° S of latitude within GBR surface waters. Qualitative N_2_ fixation rates were found to be variable across the GBR but were relatively high in coastal, inner and outer GBR waters, reaching 68 nmol L^-1^ d^-1^. Diazotroph assemblages, identified by amplicon sequencing of the *nifH* gene, were dominated by the cyanobacterium *Trichodesmium erythraeum*, γ-proteobacteria from the Gamma A clade, and δ-proteobacterial phylotypes related to sulfate-reducing genera. However, diazotroph communities exhibited significant spatial heterogeneity, correlated with shifts in dissolved inorganic nutrient concentrations. Specifically, heterotrophic diazotrophs generally increased in relative abundance with increasing concentrations of phosphate and N, while *Trichodesmium* was proportionally more abundant when concentrations of these nutrients were low. This study provides the first in-depth characterization of diazotroph community composition and N_2_ fixation dynamics within the oligotrophic, N-limited surface waters of the GBR. Our observations highlight the need to re-evaluate N cycling dynamics within oligotrophic coral reef systems, to include diverse N_2_ fixing assemblages as a potentially significant source of dissolved N within the water column.

## Introduction

The Great Barrier Reef (GBR), situated within the tropical waters of north-eastern Australia, is the largest continuous coral reef in the world and a region of high biological productivity (Furnas, 2003). Forming a natural barrier between coastal waters and the oligotrophic Coral Sea, the GBR is a biologically and biogeochemically dynamic system that is influenced by both localized hydrodynamic features (e.g., riverine discharge) (Devlin and Brodie, 2005; Waterhouse et al., 2012) and large-scale oceanographic processes (e.g., Coral Sea inflow and upwelling events) (Furnas and Mitchell, 1996; Brinkman et al., 2002; Choukroun et al., 2010).

Concentrations and sources of particulate and dissolved nutrients in GBR waters vary across spatial gradients, such as between inshore and offshore regions (Furnas et al., 1995, 2011), but bioavailable forms of dissolved inorganic nutrients are generally low (<0.05 μM) (Furnas et al., 2011). Excess concentrations of phosphate compared to dissolved inorganic nitrogen (N:P, 1–3.5) indicate that nitrogen (N) could be a limiting nutrient for phytoplankton growth (Furnas et al., 2011). Indeed, NH_4_^+^ and NO_3_^-^ stocks are typically turned over by phytoplankton within a matter of hours (Furnas et al., 2005). However, while low dissolved inorganic N to chlorophyll *a* ratios indicate that phytoplankton growth cannot be supported for more than one doubling of biomass, the measured growth rates of phytoplankton populations across the reef are paradoxically high (Furnas et al., 2005). This discrepancy between bioavailable N and phytoplankton growth rate suggests that additional N sources play a significant role in supporting the phytoplankton assemblages within the pelagic waters of the GBR. One such N source could be provided by the activity of dinitrogen (N_2_) fixing bacteria (diazotrophs).

Diazotroph activity is known to be an important source of bioavailable N within a number of discrete habitats in coral reefs systems. For example, N_2_ fixing lineages of proteobacteria and cyanobacteria are known constituents of the coral holobiont (Lema et al., 2012, 2014; Santos et al., 2014; Zhang et al., 2016), supplying fixed N to symbiotic *Symbiodinium* (Lesser et al., 2007; Ceh et al., 2013). N_2_ fixation has also been identified as an important process within coral reef sediments, contributing significantly toward NH_4_^+^ pools within upper sediment layers (Capone et al., 1992; Alongi et al., 2006). In addition, particularly high rates of N_2_ fixation, attributable to the cyanobacterium *Calothrix*, have been reported within microbial mats on intertidal reef flats (Wiebe et al., 1975; Larkum et al., 1988).

In addition to symbiotic and benthic N_2_ fixation, pelagic diazotrophic cyanobacteria have also been shown to be abundant (Bell et al., 1999; Biegala and Raimbault, 2008) and active (Hewson et al., 2007) in the water column of coral reef lagoons. An earlier study of N_2_ fixation by the photoautotrophic cyanobacterium *Trichodesmium* in northern GBR waters suggested its potential importance in the provision of fixed N to primary production (Bell et al., 1999). However, within the Heron Island Lagoon on the GBR, Hewson et al. (2007) demonstrated for bacterioplankton possessing the *nifH* gene, which encodes a subunit of the dinitrogenase reductase enzyme, diazotrophs were most similar to microbial mat and sediment-associated cyanobacteria and proteobacteria. While *Trichodesmium* and other typically planktonic phylotypes were only detected within *nifH* transcripts, suggesting they may be active but rare members of the pelagic diazotrophic assemblage in this region (Hewson et al., 2007).

While the significance of N_2_ fixation for providing bioavailable N to coral reef ecosystems has been demonstrated by the quantitative incorporation of sedimentary and benthic reef flat N_2_ fixation rates into a GBR N budget, the contribution of pelagic N_2_ fixation to GBR N cycling is less well-understood (Furnas et al., 2011). The limited available information on the diversity, abundance and activity of diazotrophic bacteria within pelagic GBR environments has hindered efforts to develop a complete N budget for the GBR (Furnas et al., 2011). To address this gap, we measured N_2_ fixation rates and determined diazotroph community composition and abundance within GBR surface waters. Further, we investigated relationships between the observed spatial patterns in diazotroph assemblage structure and the prevailing biotic and abiotic environmental characteristics across the GBR.

## Materials and Methods

### Sample Collection

Sampling was conducted during the Austral winter (6–18th July 2014), on a research voyage aboard the R/V *Cape Ferguson* (Australian Institute of Marine Science cruise 5913). The Austral winter coincides with the tropical dry season for the GBR, during which time GBR waters are generally characterized by reduced concentrations of dissolved inorganic N, phosphorous, and chlorophyll *a*, and consequently lower rates of primary production compared to Austral summer, the tropical wet season (Furnas et al., 2005).

Seawater samples were collected using 10 L Niskin bottles mounted to a hydrographic wire from sub-surface waters (5 m), to ensure that only pelagic diazotrophs were sampled while avoiding benthic contamination. Temperature, salinity and chlorophyll fluorescence were determined using a Seabird SEB19+ Conductivity-Temperature-Depth recorder. Raw chlorophyll fluorescence readings from the CTD (Wetlabs Wetstar chlorophyll fluorometer) were empirically calibrated to *in situ* chlorophyll *a* (μg L^-1^) by building a calibration regression between fluorescence and discrete chlorophyll measurements from Niskin samples.

### Dissolved Inorganic Nutrient Analyses

Samples for dissolved inorganic nutrient analyses, including NO_x_ (NO_3_^-^ + NO_2_^-^), PO_4_^3-^ and SiO_4_^4-^ (45 ml) were passed through a 0.45 μm (Filtropur, Sarsedt) syringe filter, collected in 50 ml Falcon tubes and stored at -20°C. Concentrations of NO_x_, PO_4_^3-^, SiO_4_^4-^, were determined on a Flow Injection Analyzer (Lachat QuikChem 8000) at the Office for Environment and Heritage (Sydney, NSW, Australia), with a limit of detection of 0.01 μM. In addition, ammonium concentrations were analyzed at sea immediately after collection using the OPA fluorometric method (Holmes et al., 1999).

### Flow Cytometric Analyses

Triplicate 1 ml samples for microbial cell enumeration using flow cytometry (FCM) were fixed with glutaraldehyde (2% final concentration), snap frozen and stored in liquid nitrogen on-board, prior to -80°C storage post-voyage. Prior to FCM analysis, samples were quick-thawed and divided to enable the separate enumeration of bacteria (200 μl) and autofluorescent picophytoplankton (800 μl). Samples for bacterial enumeration were stained with SYBR Green I [1:10,000] (Invitrogen Molecular Probes, United States), while picophytoplankton samples were analyzed unstained. For both sample types, 1 μm diameter fluorescent microspheres (Invitrogen Molecular Probes) were added as an internal reference (Marie et al., 1997; Gasol and del Giorgio, 2000). Samples were analyzed using a Becton Dickinson LSR II flow cytometer (BD Biosciences), with bacteria discriminated according to SYBR Green fluorescence and side-scatter, while picophytoplankton populations were discriminated according to orange (phycoerthyrin) fluorescence, red (chlorophyll *a)* fluorescence and side-scatter (Seymour et al., 2007). All data were analyzed using Cell-Quest Pro software (BD Biosciences).

### Dinitrogen Fixation Incubation

For quantification of particulate carbon and N concentrations, and natural abundance stable isotope analyses (δ^15^N, T_0_ for N_2_ fixation incubations), between 1 and 4 L of seawater was filtered onto a pre-combusted glass fiber filter (GF/F, 0.7 μm pore size, Whatman, United Kingdom), and stored at -20°C. Post-voyage, natural abundance filters were dried at 60°C for 48 h before being analyzed on an elemental analyzer (Thermo Finnigan MAT Conflo IV) coupled to an isotope ratio mass spectrometer (Thermo Finnigan Delta XP; limit of detection = 15 μg N per filter) (Research Corporation of the University of Hawaii).

At each station, triplicate 4 L polycarbonate, HCl clean Nalgene incubation bottles were filled via silicone tubing directly from Niskin bottles. Bottles were capped with septa without introducing headspace, then injected with 3 ml ^15^N_2_ gas (98 atom%, Sigma–Aldrich, Australia) and inverted 100 times, leading to a theoretical enrichment of 7–8 atom%, assuming complete dissolution of the ^15^N_2_ gas bubble (Montoya et al., 1996). Efforts were made to ensure that all injections occurred during the middle of the light period (approximately between 10 am and 2 pm). Bottles were incubated in deck-board incubators filled with continuously flowing surface sea water and shaded with Lee Filters 061 Mist Blue filter (Andover, United Kingdom) to replicate *in situ* light levels. After ∼24 h, incubations were terminated by filtration onto pre-combusted GF/F (0.7 μm pore size, Whatman) and frozen at -20°C.

Post-voyage, enriched filters were dried separately from the natural abundance filters at 60°C for 48 h, and isotopic composition along with total particulate N and carbon were determined using an elemental analyzer (Thermo Finnigan MAT Conflo IV) coupled to an isotope ratio mass spectrometer (Thermo Finnigan Delta XP; limit of detection = 15 μg N per filter) (Research Corporation of the University of Hawaii). Volumetric assimilation rates were calculated as previously described (Montoya et al., 1996), using a corrected atom% enrichment value of 75% of the theoretical (Mohr et al., 2010), and are considered qualitative due to the known incomplete dissolution of the ^15^N_2_ gas bubble (Mohr et al., 2010; Großkopf et al., 2012).

### DNA Collection and Extraction

Triplicate 2–4 L seawater samples were immediately filtered onto 0.2 μm membrane filters (Durapore, Merck Millipore) and stored at -20°C on-board (1–12 days), before being stored at -80°C post-voyage. Microbial community DNA was extracted from preserved filters using the PowerWater DNA Extraction Kit (MoBio Laboratories, Carlsbad, CA, United States) according to the manufacturer’s instructions, with the exception of an additional 10 min heating step with solution PW1 to 60°C prior to 10 min of bead beating, to ensure complete cell lysis. DNA yield was quantified using a Broad Range DNA Qubit^TM^ Assay (Invitrogen, Thermo Fisher Scientific, Scoresby, VIC, Australia) with a Qubit^TM^ 2.0 Fluorometer.

### Amplicon Sequencing Analysis

The composition of the diazotrophic assemblage at each site was determined using a nested PCR protocol targeting a 327 base pair region of the *nifH* gene for biological replicates (*n* = 3) pooled in equal volumes. The two sets of degenerate primers included the nifH3 (5′-ATRTTRTTNGCNGCRTA-3′) reverse and nifH4 (5′-TTYTAYGGNAARGGNGG-3′) forward primer pair, followed by the nifH1 (5′-TGYGAYCCNAARGCNGA-3′) forward and the nifH2 reverse (5′-ADNGCCATCATYTCNCC-3′) primer pair (Zehr and McReynolds, 1989; Zehr and Turner, 2001). PCR was performed using the following conditions: 95°C (2 min) initial denaturation and 30 cycles of 95°C denaturation (1 min), 48°C annealing (1 min) and 72°C extension (1 min), followed by a final extension at 72°C (10 min). The nucleotide composition of *nifH* amplicons were identified using 454 pyrosequencing (Roche, FLX Titanium; Molecular Research LP) after an additional 10 PCR cycles with custom barcoded nifH1 and nifH2 primers under the same reaction conditions (Dowd et al., 2008; Messer et al., 2015a), with between 5110 and 10860 sequences retrieved per sample (3561–7939 high quality sequences; Supplementary Table 1). These sequences have been submitted to the Sequence Read Archive under accession numbers SRR3502520- SRR3502530.

The open source software “Quantitative Insights into Microbial Ecology” (QIIME) (Caporaso et al., 2010a) was used to analyze and process amplicon sequencing data. Raw *nifH* sequences were quality filtered, such that sequences with a quality score <25 and reads <200 base pairs in length were removed and subject to reference-based and *de novo* chimera removal using USEARCH61 with default parameters (Edgar, 2010). The reference database for chimera removal comprised unaligned *nifH* sequences exported from a custom *nifH* database (Zehr et al., 2003; Heller et al., 2014). Sequences were then clustered into operational taxonomic units (OTUs) at 97% nucleotide sequence identity using UCLUST, whereby *nifH* sequences within 3% of the most abundant read were assigned as OTUs (Edgar, 2010). An OTU by sample table was generated and filtered to remove low abundance OTUs (<50 sequences in total), then rarefied to the lowest number of sequences per sample (1394 sequences), resulting in a total of 92 OTUs. The FrameBot tool from the FunGene pipeline was used to identify any stop codons and correct frameshifts, and to simultaneously assign taxonomy based on amino acid identity (AAI) and alignment of the 92 OTUs to the Ribosomal Database Project *nifH* database (Fish et al., 2013). The PyNAST (Caporaso et al., 2010b) tool was then used with default parameters to BLAST and align representative nucleotide sequences from *nifH* OTUs to the closest *nifH* sequence in an aligned custom *nifH* database (exported from Arb) (Zehr et al., 2003; Heller et al., 2014). A maximum likelihood phylogenetic tree was generated from aligned OTUs (92 sequences) and publically available *nifH* sequences (121 nucleotide sequences) using the Tamura-Nai model in MEGA (v7.0) (Tamura and Nei, 1993; Kumar et al., 2016).

### Quantitative PCR (qPCR) Assays

The two most abundant *nifH* clades observed in the amplicon sequencing analysis, representing *Trichodesmium* spp. and the Gamma A clade, were quantified directly using previously designed Taqman qPCR primers and probes (**Table 1**). These established qPCR assays were chosen because they targeted the dominant *Trichodesmium* and Gamma A OTUs from our amplicon pyrosequencing analyses. Specifically, 3 OTUs in our dataset (out of 92) shared 100% identity between the forward primer, probe, and reverse primer designed to quantify *Trichodesmium* spp. by Church et al. (2005), including OTU5947, OTU3248, and OTU6010. While 6 OTUs (out of 92) shared 100% identity between the Gamma A forward primer, probe, and reverse primer, including OTU2275, OTU412, OTU4346, OTU481, OTU5337, OTU5802 of the Moisander et al. (2008, 2014) assay.

**Table 1:** Quantitative PCR primers, probes and reaction conditions for two *nifH* OTUs targeted during this study.

Target	Forward primer	Reverse primer	TaqMan probe	Reaction conditions
Tricho.^a^	GACGAAGTATTGAAGCCAGGTTTC	CGGCCAGCGCAACCTA	CATTAAGTGTGTTGAATCTGGTGGTCCTGAGC	50°C (5 min)
				95°C (10 min)
				40 cycles:
				95°C (15 s)
Gamma A^b^	CGGTAGAGGATCTTGAGCTTGAA	CACCTGACTCCACGCACTTG	AAGTGCTTAAGGTTGGCTTTGGCGACA	60°C (60 s)

*^a^Church et al., 2005; ^b^Moisander et al., 2008, 2014. All probes are 5′-FAM (dye) and TAMRA-3′ (quencher).*

In order to generate qPCR standards, taxon specific PCR primers were used to amplify a fragment of the *nifH* gene target and the resultant product was cloned into a P-Gem T Easy Vector (Promega, Sydney, NSW, Australia) and transformed into competent TOPO *Escherichia coli* cells (Thermo Fisher Scientific, Scoresby, VIC, Australia). Following overnight growth at 37°C on LB agar plates containing ampicillin [50 mg/ml] and IPTG/X-gal, plasmids were extracted and purified from white colonies using the Plasmid Mini Kit (Bioline, Sydney, NSW, Australia). Confirmation of the correct *nifH* gene insert was completed using Sanger sequencing at the Australian Genome Research Facility. All *nifH* standards were serially diluted in sterile nucleic-acid-free H_2_O and a standard curve with concentrations ranging from 10^2^ to 10^7^ *nifH* copies was run alongside all samples, along with no template (negative) controls containing 5 μl of nucleic-acid-free H_2_O. To prevent inhibition of qPCR assays, template DNA was diluted 1/5 using nucleic-acid-free H_2_O. Following this, 5 μl of the template dilution was used in the 20 μl qPCR assays. Each qPCR reaction also included 200 nM of each primer, 100 nM probe, 2x TaqMan Master Mix II, 3 μl of nucleic-acid-free H_2_O. qPCR assays were run on triplicate biological replicates, including triplicate technical replicates for each sample and standard, using previously described reaction conditions (**Table 1**) (Church et al., 2005; Moisander et al., 2008, 2014) using a StepOnePlus^TM^ Real-Time PCR machine (Applied Biosystems, Thermo Fisher Scientific, Scoresby, VIC, Australia). Linear regression analyses of quantification cycle (Cq) versus log10 *nifH* gene copies were conducted using the StepOnePlus^TM^ software (v2.3), and demonstrated that our *Trichodesmium* assay had a mean *R*^2^ of 0.999 and a reaction efficiency of 100.01% (*n* = 3), while the Gamma A assay had a mean *R*^2^ of 0.996 and a reaction efficiency of 96.60% (*n* = 3). The Cq limit for each assay was between 35 and 36 cycles (out of 40) equivalent to a detection of ∼5–6 *nifH* copies per reaction.

### Statistical Analyses

Distance-based linear modeling (distLM) was used in order to identify relationships between environmental parameters and spatial heterogeneity (dissimilarity between sites) in diazotroph community composition (determined by amplicon sequencing) across the GBR. DistLM was performed on a square-root transformed Bray–Curtis dissimilarity matrix of 92 *nifH* OTUs, and standardized log transformed environmental parameters in the PRIMER + PERMANOVA software (v7, Clarke and Gorley, 2015). To identify relationships between the abundance of *Trichodesmium* spp. and the Gamma A clade (determined by qPCR), Pearson correlation coefficients were calculated between environmental parameters, and total bacterial and phytoplankton abundances (determined by flow cytometry) in Minitab (v17).

## Results

### Abiotic and Biotic Characteristics of GBR Surface Waters

**FIGURE 1:**
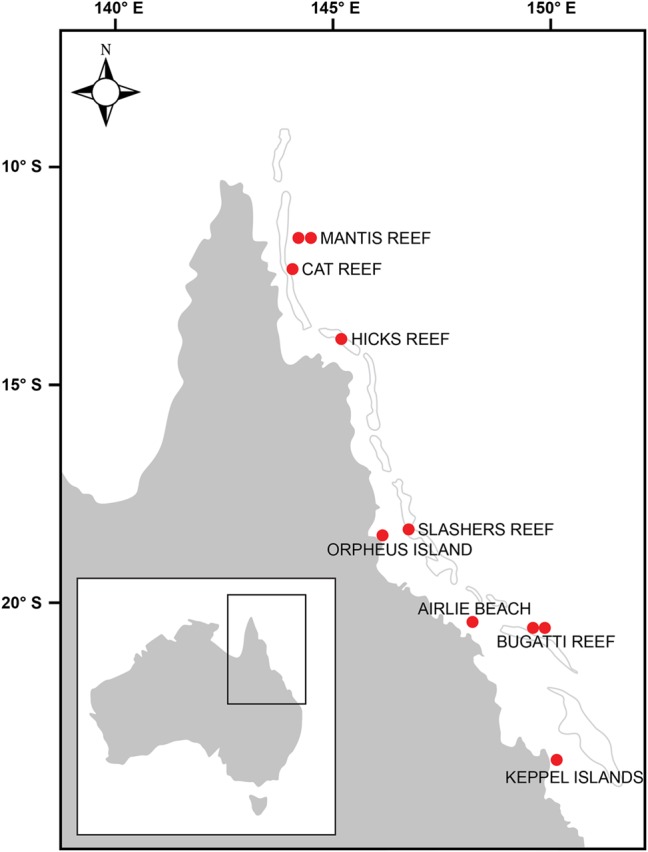
Map of the Great Barrier Reef (GBR) demonstrating the 10 locations where samples were collected from surface waters (5 m) during this study.

**FIGURE 2:**
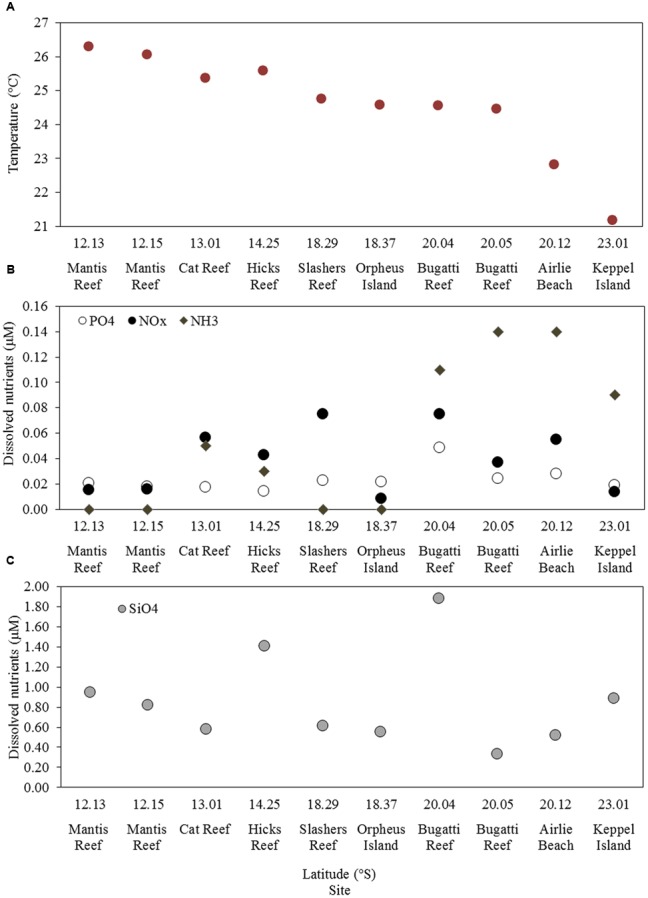
Environmental characteristics of each study site including: **(A)** sea surface temperature (SST), **(B)** concentrations of NH_3_, PO_4_ and NOx, **(C)** concentrations of SiO_4_.

Samples were collected at 10 stations located between latitudes 12° S (northern GBR) and 23° S (southern GBR) and encompassed a variety of regions including coastal, central and outer GBR waters (**Figure 1** and Supplementary Figure 1). Sea surface temperature (SST), salinity, and dissolved inorganic nutrient concentrations at the time of sampling were generally characteristic of the tropical oligotrophic conditions that prevail across most of the GBR (**Figure 2** and **Table 2**). However, significant spatial heterogeneity in some of these environmental variables was observed across the 10 sampling sites. While salinity was relatively consistent, with a mean (± standard deviation) of 35.2 ± 0.2 PSU, SST varied with latitude between 21.2 and 26.3°C, with a mean of 24.6 ± 1.5°C (**Figure 2A**). Mean chlorophyll *a* concentrations at the 5 m sampling depth were 0.34 ± 0.15 μg L^-1^ and varied substantially from 0.08 μg L^-1^ at the inner Mantis Reef site to 0.57 μg L^-1^ at Cat Reef (both northern GBR; **Figure 1** and **Table 2**). Dissolved inorganic nutrients were generally low, with mean ammonia (NH_3_), silicate (SiO_4_^4-^), phosphate (PO_4_^3-^), and oxides of nitrogen (NO_x_ = NO_3_^-^ + NO_2_^-^) concentrations of 0.06 ± 0.06, 0.85 ± 0.47, 0.02 ± 0.01, and 0.04 ± 0.03 μM respectively. SiO_4_^4-^ concentrations were the most variable, for example ranging from 0.33 to 1.88 μM at the inner and outer Bugatti Reef sites (southern GBR) respectively (**Figure 2**). In general, NH_3_ concentrations increased with latitude, ranging from below detection (displayed as 0.00 μM) at outer Mantis Reef (northern GBR) to 0.14 μM in the coastal waters of Airlie Beach (southern GBR) (**Figure 2**).

**Table 2:** Locations of sampling stations shown in **Figure 1** with environmental and contextual meta-data, including: salinity, chlorophyll *a* (Chl*a*.), and cell counts determined by flow cytometry for total bacteria, *Synechococcus* (Syne.), *Prochlorococcus* (Proc.), and picoeukaryote (Pico.) populations.

Station	Location	Lat. (°S)	Long. (°E)	Bottom depth (m)	Salinity (PSU)	Chl*a*. (μg L^-1^)	Total bacteria (cells ml^-1^)	Syne. (cells ml^-1^)	Proc. (cells ml^-1^)	Pico. (cells ml^-1^)
CSC82	Mantis Reef	12.13	143.52	270	35.09	0.31	9.4 × 10^5^	9.8 × 10^4^	1.1 × 10^5^	5.0 × 10^3^
CSC85	Mantis Reef	12.15	143.53	31	35.07	0.08	3.5 × 10^5^	1.0 × 10^4^	3.6 × 10^4^	1.4 × 10^3^
CSC88	Cat Reef	13.01	143.49	23	34.8	0.57	nd	nd	nd	nd
CSC89	Hicks Reef	14.25	145.26	300	35.04	0.21	5.5 × 10^5^	6.2 × 10^4^	9.1 × 10^4^	6.6 × 10^3^
CSC93	Slashers Reef	18.29	147.01	45	35.16	0.3	1.2 × 10^6^	8.1 × 10^4^	1.1 × 10^5^	1.3 × 10^4^
CSC81	Orpheus Island	18.37	146.28	24	35.37	0.43	1.2 × 10^6^	2.1 × 10^5^	3.8 × 10^4^	6.5 × 10^3^
CSC97	Bugatti Reef	20.04	150.17	65	35.2	0.31	8.0 × 10^5^	5.6 × 10^4^	4.8 × 10^4^	5.4 × 10^3^
CSC96	Bugatti Reef	20.05	150.18	18	35.21	0.43	8.1 × 10^5^	7.3 × 10^4^	6.7 × 10^4^	4.4 × 10^3^
CSC94	Airlie Beach	20.12	148.44	26	35.14	0.51	1.1 × 10^6^	6.9 × 10^4^	3.4 × 10^4^	7.4 × 10^3^
CSC98	Keppel Island	23.01	150.53	21	35.53	0.24	1.2 × 10^6^	2.0 × 10^5^	9.7 × 10^4^	1.0 × 10^4^

We observed clear differences in the abundances of total bacteria, photosynthetic bacterioplankton and photosynthetic picoeukaryotes across the GBR (**Table 2**). For example, low abundances of all populations were observed at inner Mantis Reef in the northern GBR, while high abundances of all populations were observed in the coastal waters at Keppel Islands in the southern GBR (**Table 2**). In addition, higher abundances of bacteria (1.2 ± 0.04 × 10^6^ cells ml^-1^) and *Synechococcus* (2.1 ± 0.2 × 10^5^ cells ml^-1^) were observed at Orpheus Island (central GBR), and higher abundances of bacteria (1.2 ± 0.08 × 10^6^ cells ml^-1^), *Prochlorococcus* (1.1 ± 0.05 × 10^5^ cells ml^-1^) and picoeukaryotes (1.3 ± 0.4 × 10^4^ cells ml^-1^) were observed at Slashers Reef (central GBR; **Table 2**).

### Rates of N_2_ Fixation in GBR Waters

**FIGURE 3:**
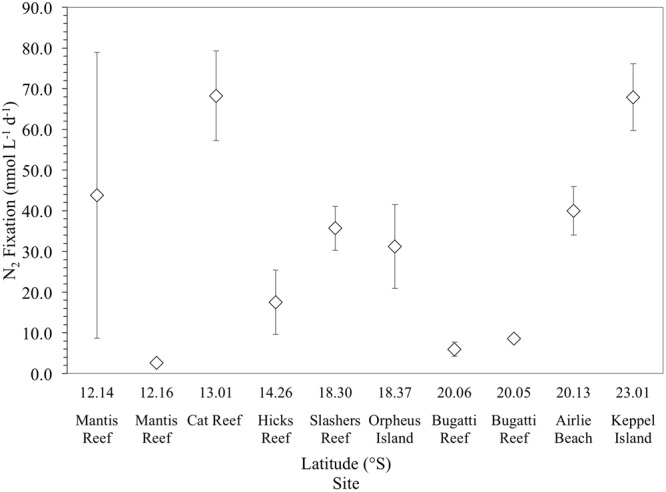
Mean N_2_ fixation rates (nmol L^-1^ d^-1^) of triplicate incubations (corrected for the incomplete dissolution of ^15^N_2_ in seawater). Error bars represent the standard deviation about the mean.

Mean qualitative N_2_ fixation rates across the 10 sampling sites were 32 ± 24 nmol N L^-1^ d^-1^ (*n* = 30). N_2_ fixation rates were highly variable, ranging from a minimum of 3 ± 0.8 nmol N L^-1^ d^-1^ at the inner Mantis Reef site, to a maximum of 68 ± 11 nmol N L^-1^ d^-1^ at Cat Reef (both northern GBR). Comparatively high mean rates of N_2_ fixation (≥30 nmol N L^-1^ d^-1^) were observed at 6 out of the 10 sampling sites (**Figure 3**), while comparatively low rates (≤9 nmol N L^-1^ d^-1^) were measured at three sites, including inner Mantis Reef in the north, and inner and outer Bugatti reef, southern GBR (**Figure 3**).

### Diazotroph Diversity and Abundance Across the GBR

**FIGURE 4:**
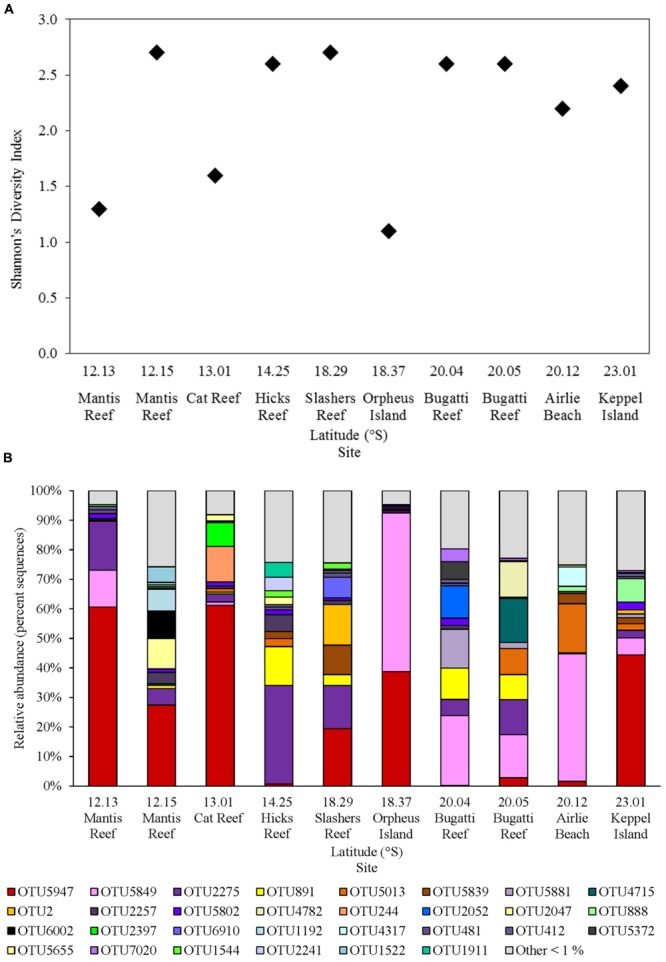
Diazotroph assemblages within GBR surface waters, including **(A)** *nifH* diversity (Shannon’s index) of rarefied sequence data and **(B)** relative abundance of *nifH* OTUs (% of total sequences) clustered at 97% sequence similarity, with OTUs representing <1% of total sequences grouped as “Other <1%.”

The *nifH* gene fragment was amplified from all sites within GBR waters, resulting in a total of 92 unique *nifH* OTUs at 97% nucleotide identity, and between 15 and 37 OTUs per sample, after quality filtering and removal of low abundance OTUs (<50 total sequences; Supplementary Table 1). The highest levels of diazotroph diversity (Shannon’s diversity index, H′ = 2.7) occurred at the inner Mantis Reef site in the north and Slashers Reef in the central GBR. While the lowest levels of diversity (H′ < 1.3) were observed at Orpheus Island in the central GBR and at the outer Mantis Reef site in the northern GBR (**Figure 4A**).

**FIGURE 5:**
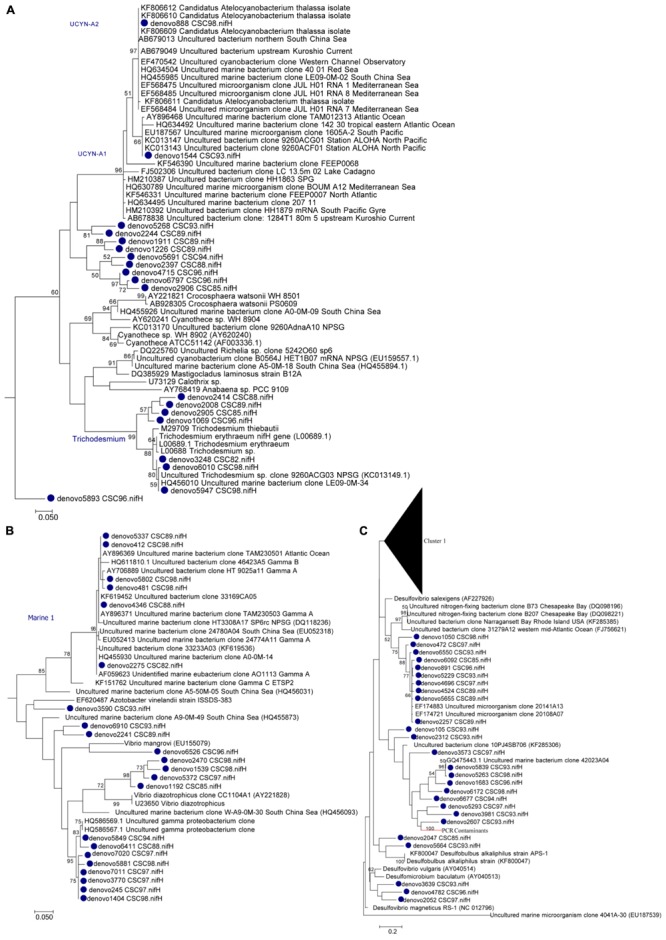
Maximum likelihood phylogenetic subtree of **(A)** Cluster IB Cyanobacteria, **(B)** Cluster IG Proteobacteria, and **(C)** Cluster III *nifH* OTUs (clustered at 97% nucleotide identity). Bootstrap support ≥50% is shown for 1000 bootstraps. Please refer to Supplementary Figure 2 for the full *nifH* phylogenetic tree.

**FIGURE 6:**
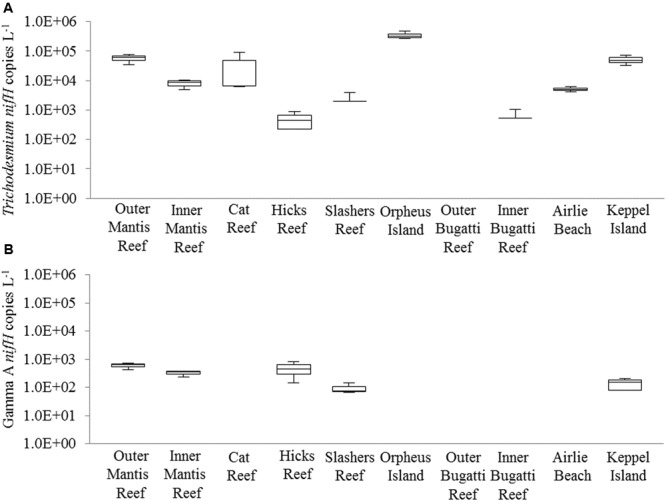
Quantitative PCR (qPCR) abundances (*nifH* copies L^-1^) of **(A)** *Trichodesmium* and **(B)** the Gamma A clade across GBR sampling locations (from northern to southern waters). The box and whiskers represent the maximum value, 75th percentile, median value (50th percentile), 25th percentile, and the minimum value for the triplicate biological replicates. Where no box is present, genes were detected but were below the limit of quantification and were therefore given values of “0.” Please note the y-axis is shown on the log scale for clarity.

Diazotrophic populations across the GBR included a range of OTUs that displayed sequence similarities to known Cluster IB photoautotrophic and photoheterotrophic cyanobacteria, as well as a number of Cluster IG and Cluster III proteobacterial diazotrophs (**Figure 5** and Supplementary Figure 2). The most abundant OTU in the dataset was OTU5947, which shared 95% AAI with the filamentous cyanobacterium *Trichodesmium erythraeum*, and clustered with representative and environmental *Trichodesmium* sequences (**Figure 5A**). OTU5947 represented 27% of total *nifH* sequences. This *Trichodesmium* OTU was present at each of the 10 sampling sites, from northern to southern waters, including coastal, inner reef and outer GBR locations, where maximum relative abundances of 44, 19, and 61% of *nifH* sequences occurred respectively (**Figure 4B**). Patterns in the *Trichodesmium*-specific qPCR analyses targeting OTU5947, OTU3248, and OTU6010, corresponded to those observed with the amplicon sequencing profiles, whereby the maximum abundance of *Trichodesmium nifH* copies L^-1^ occurred in the central GBR at Orpheus Island, at the outer Mantis Reef site in the north, and at Keppel Islands in the south, with mean abundances of 3.5 × 10^5^, 5.7 × 10^4^, and 5.2 × 10^4^ L^-1^, respectively (**Figure 6**).

Relative to *Trichodesmium*, other Cluster IB cyanobacterial *nifH* OTUs occurred less frequently in the dataset. For example, OTU4715, which shared 88% AAI with the filamentous cyanobacterium *Leptolyngbya* spp., contributed only 2% to the total number of *nifH* sequences and was restricted to 2 out of 10 sites, where it comprised a maximum relative abundance of 15% of the diazotroph assemblage (Bugatti Reef; **Figure 4B**). Two other cyanobacterial OTUs, OTU888 and 1544, which shared 95 and 96% AAI respectively with the unicellular cyanobacterium *Candidatus* Atelocyanobacterium thalassa (UCYN-A), also comprised only ∼2% of total *nifH* sequences. Interestingly, OTU888 clustered with representative sequences from the UCYN-A2 ecotype, while OTU1544 was more closely related to the UCYN-A1 ecotype (**Figure 5A**). These two OTUs were present at two and four sites respectively, but did not make up more than 8% of the diazotroph assemblage when detected (e.g., Keppel Island, southern GBR; **Figure 4B**).

Outside of Cluster IB, significant numbers of *nifH* sequences associated with putative heterotrophic diazotrophs were detected, including representatives of the γ-proteobacteria from Cluster IG (**Figure 5B** and Supplementary Figure 2). The dominant heterotrophic *nifH* sequences were associated with the OTUs 5849 and 2275, which shared 91 and 88% AAI with the γ-proteobacterium *Pseudomonas stutzeri*. Collectively, these γ-proteobacterial OTUs comprised 26% of total *nifH* sequences and, at their most abundant, made up 54 and 34% of the diazotroph assemblage at Orpheus Island (central GBR) and Hicks Reef (northern GBR) respectively (**Figure 4B**). One of these two OTUs, OTU2275 clustered with the Gamma A clade (**Figure 5B**), within the Marine 1 group (Langlois et al., 2015). qPCR analyses revealed that the Gamma A clade reached a maximum mean abundance of 5.9 × 10^2^ *nifH* copies L^-1^ at the outer Mantis Reef site, in the northern GBR, but was not detectable at 5 out of 10 sites (**Figure 5B**). In addition, the Gamma A clade was typically found to be between 1 and 5 orders of magnitude less abundant than *Trichodesmium*, except at the Hicks Reef site in the north, where mean abundances of both taxa were ∼4.5 × 10^2^ *nifH* copies L^-1^ (**Figure 5**).

Alongside the γ-proteobacterial *nifH* sequences, a number of sequences that were closely related to sulfate-reducing genera of the δ-proteobacteria from Cluster III were also frequently detected, collectively comprising 17% of total *nifH* sequences (**Figures 4B, 5C**). In particular, three OTUs (OTU891, 2257, and 5655) most closely related to members of *Desulfovibrio* spp. (90% AAI) comprised 6% of total *nifH* sequences. OTUs 881 and 2257 were relatively widespread across the GBR, being present at eight and seven of the sampling sites respectively, comprising up to 13% of the diazotroph community at Hicks Reef, northern GBR (**Figure 4B**).

### Diazotroph Community Composition Correlates to PO_4_ and DIN Concentrations

Distance-based linear modeling identified PO_4_ and dissolved inorganic N (DIN) concentrations, as the measured environmental variables that were significant (*P* < 0.05) predictors of spatial heterogeneity in diazotroph community composition (Supplementary Table 2). Sites with higher PO_4_ and DIN concentrations, such as Hicks Reef in the north, outer and inner Bugatti Reef in the south, and the coastal waters of Airlie Beach, southern GBR (**Figure 2** and Supplementary Figure 3), contained lower relative abundances of *Trichodesmium nifH* sequences, but higher relative abundances of the γ and δ-proteobacterial OTUs (**Figure 4**). However, no significant Pearson correlations were observed between the absolute abundances (derived by qPCR) of the *Trichodesmium* and Gamma A groups and any of the environmental parameters. In addition, despite evidence of variation in chlorophyll *a* concentrations across the sampling sites (**Table 2**), indicative of changing phytoplankton biomass, no significant associations were observed between chlorophyll *a* and diazotroph community composition.

## Discussion

The paradoxical nature of many coral reefs, whereby relatively high biological productivity occurs within marine waters where ambient concentrations of dissolved inorganic nutrients are low, has resulted in efforts to reconcile nutrient dynamics within coral reef systems (e.g., Furnas et al., 1995, 2005, 2011; Hearn et al., 2001; Falter et al., 2004; Schaffelke et al., 2012). Some early studies demonstrated that biological N_2_ fixation might play an important role in supplying bioavailable N within benthic reef habitats (Webb et al., 1975; Wiebe et al., 1975; Larkum et al., 1988), and more recently, diazotrophic bacteria have been shown to be an important constituent of the coral holobiont, supplying N requirements for symbiotic *Symbiodinium* (Lesser et al., 2004, 2007; Lema et al., 2012, 2014; Olson and Lesser, 2013; Zhang et al., 2016). However, within the pelagic environment of the GBR the role of biological N_2_ fixation is less well-understood (Furnas et al., 2011), despite evidence to suggest that large discrepancies exist between nutrient availability and phytoplankton growth (Furnas et al., 2005). Here, we found a diverse community of cyanobacterial and non-cyanobacterial diazotrophs inhabiting GBR surface waters during Austral winter, and relatively high qualitative rates of N_2_ fixation within coastal, inner and outer reef habitats, indicating that diazotrophic bacterioplankton might act as a significant source of fixed N within the oligotrophic GBR.

Through our *nifH* amplicon sequencing analyses, we provide the first in-depth characterization of the potential for N_2_ fixation within bacterioplankton assemblages across the GBR. Using this approach, Cluster 1B cyanobacterial diazotrophs were identified as the dominant phylotype distributed throughout GBR surface waters, specifically OTUs closely related to *Trichodesmium* spp. showed high relative and absolute abundances. Seven OTUs sharing >90% AAI to *Trichodesmium erythraeum* were detected in the amplicon sequencing dataset, three of which were targeted by the *Trichodesmium* spp. qPCR assay employed herein. *Trichodesmium* spp. are routinely observed in tropical, oligotrophic environments, including coral reef lagoons, where they can form large surface aggregations and contribute substantially to N_2_ fixation (Bell et al., 1999; Campbell et al., 2005; McKinna et al., 2011; Bonnet et al., 2015b; Turk-Kubo et al., 2015). Although surface aggregations were not observed during the present study, qPCR analyses indicated that the abundance of *Trichodesmium* spp. were at times high, reaching up to 3.5 × 10^5^ *nifH* copies L^-1^, consistent with observations from the neighboring Coral and Solomon Seas during Austral winter conditions (Bonnet et al., 2015b). *Trichodesmium* has previously been recognized as an important feature of pelagic GBR microbial communities, through microscopic enumeration and satellite remote sensing (Bell et al., 1999; McKinna et al., 2011), and it is estimated that N_2_ fixation by *Trichodesmium* alone could contribute ∼0.7–3.0 t N km^-2^ to GBR surface waters. At sites where qualitative N_2_ fixation rates of ∼68 nmol L^-1^ d^-1^ were observed, we found *Trichodesmium* to be abundant (3.4–5.2 × 10^4^ *nifH* copies L^-1^). By virtue of both its abundance, diversity and activity, *Trichodesmium* therefore potentially plays a very important role in supporting the growth and production of non-diazotrophic assemblages across the GBR pelagic zone.

Beyond *Trichodesmium*, a number of other OTUs affiliated with Cluster 1B cyanobacterial diazotrophs were present in GBR surface waters. These included OTUs related to the filamentous genus *Leptolyngbya*, which has previously been found to actively fix N_2_ in benthic cyanobacterial mats (Woebken et al., 2014) including within coral reef systems (Charpy et al., 2010), and OTUs associated with the unicellular cyanobacterial symbiont UCYN-A, including ecotypes 1 and 2 which are widely distributed throughout the global ocean (Farnelid et al., 2016; Zehr et al., 2016). The majority of these Cluster 1B OTUs were present in relatively low abundances, contributing to <15 and 10% of *nifH* sequences at their maxima. The observed low relative abundance of UCYN-A1 and UCYN-A2 in GBR surface waters during Austral winter is consistent with our previous observations for the adjoining Coral Sea, whereby UCYN-A ecotypes effectively disappeared in Austral winter when compared to spring (Messer et al., 2015b). However, in the eastern Coral Sea, high UCYN-A abundances (determined by qPCR) have been reported during Austral winter (Bonnet et al., 2015b), and UCYN-A has been reported to be the dominant diazotrophic phylotype within the Noumea Lagoon, New Caledonia (Turk-Kubo et al., 2015). While maximum abundances of UCYN-A appear to occur at more southern latitudes in the western South Pacific during Austral autumn (Moisander et al., 2010).

In addition to the cyanobacterial diazotrophs, our data provide the first estimates of the diversity and abundance of heterotrophic diazotrophs in GBR surface waters. Of particular importance were the Gamma A OTUs affiliated with Cluster 1G, which were the second most prevalent diazotrophic phylotype in our amplicon sequencing analyses. Specifically, OTUs clustering with the Gamma A group at times comprised >50% of sequences at a given site, although the abundances of these organisms as determined by qPCR were generally low, with peaks of only 5.9 × 10^2^ *nifH* copies L^-1^. This could reflect the possible preferential amplification of this phylotype by the PCR primers used in this study (Turk-Kubo et al., 2012), and highlights the importance of complementary qPCR analyses to verify amplicon sequencing based approaches. The abundance of Gamma A throughout GBR surface waters are in-line with previous studies utilizing Gamma A qPCR assays. For instance, Bonnet et al. (2015b) reported similar Gamma A abundances in the eastern Coral Sea and Solomon Sea, and Moisander et al. (2014) reported median Gamma A abundances of 8 × 10^2^ *nifH* copies L^-1^ throughout the western South Pacific Ocean. Indeed, low abundances of Gamma A have been reported for much of the major ocean basins, indicating that they are a ubiquitous component of diazotrophic bacterioplankton in tropical, oligotrophic ecosystems (Langlois et al., 2015). Our data show the Gamma A clade to also be widespread throughout GBR surface waters, and it is particularly notable that members of this clade comprised a significant proportion of the diazotroph community at sites where relatively high qualitative rates of community N_2_ fixation were observed while *Trichodesmium* abundances were low.

Compared to the few other studies reporting water column N_2_ fixation rates in coral reef environments, the qualitative rates we observed in GBR waters were relatively high. For example, the highest rates observed here, between 31 and 68 nmol L^-1^ d^-1^, were greater than those observed in a New Caledonian coral lagoon (<10 nmol L^-1^ d^-1^; Biegala and Raimbault, 2008), substantially greater than those reported for the eastern Coral Sea (≤2 nmol L^-1^ d^-1^; Bonnet et al., 2015b), and in-line with those measured in the western Coral Sea, adjacent to the GBR (56 nmol L^-1^ d^-1^; Messer et al., 2015b). Moreover, the rates observed in the present study are within the higher range of N_2_ fixation rates compiled within a global database of marine N_2_ fixation (Luo et al., 2012), indicating that N_2_ fixation within GBR waters indeed represents a significant source of N at the local scale, and a potentially significant region of N_2_ fixation activity at the global scale.

It must be noted, however, that the “bubble” method used to measure N_2_ fixation (Montoya et al., 1996) in this study, has previously been shown to underestimate N_2_ fixation by 50% or more, and will also depend on the composition of the underlying diazotroph community, as well as the time of sampling relative to the diurnal cycle of N_2_ fixation within specific clades, and the physical properties of the sampling site (e.g., temperature and salinity) which all influence the solubility of N_2_ gas in seawater (Mohr et al., 2010; Großkopf et al., 2012; Wilson et al., 2012; Benavides et al., 2013). Despite these caveats, the Montoya et al. (1996) method was applied in this study because it was decided that it was favorable to underestimate the significance of N_2_ fixation on the GBR, rather than potentially overestimate it by inadvertently introducing additional particulates, nutrients, or trace metals through pre-preparing ^15^N enriched natural or artificial seawater. Indeed, a recent study demonstrated that the preparation of ^15^N_2_ enriched seawater could result in the enrichment of trace metals by up to 0.1 nmol L^-1^, due to contact with standard laboratory ware used to prepare the solution (glass, rubber, and plastic) (Klawonn et al., 2015). Given that samples collected for each incubation experiment had distinct physical and chemical properties, such as variability in salinity and dissolved inorganic nutrient concentrations (**Table 2**), introducing enriched seawater that did not match the properties of the coastal, inner and outer GBR seawater sampled, could have influenced nutrient dynamics within our incubations. Therefore, our rate calculations were corrected according to Mohr et al. (2010) to account for the incomplete dissolution of ^15^N_2_ in seawater.

In addition, some commercially available ^15^N_2_ gas stocks have recently been found to be contaminated with ^15^NO_3_, ^15^NH_4_, and ^15^N_2_O (Dabundo et al., 2014). Our study was performed prior to the publication of the Dabundo et al. (2014) study which reported the contamination of two batches of Sigma–Aldrich ^15^N_2_ gas stocks, and Sigma–Aldrich gas lot SZ1670V (2013 batch) was used in this study. We cannot explicitly rule out that there was not contamination in the batch of ^15^N_2_ that we used, therefore using the average concentration of ^15^NO_3_, ^15^NH_4_, and ^15^N_2_O contamination in Sigma–Aldrich stocks reported by Dabundo et al. (2014) (298, 818, and 61 μmol/mole ^15^N respectively) we calculated that only an additional 3.2 × 10^-7^ moles of ^15^N could have been added to our incubations during our trace additions (2.7 × 10^-4^ moles) of ^15^N_2_ gas (Supplementary Table 3). Therefore, we found that any potential ^15^N contamination would have had a negligible effect on our measured rates of N_2_ fixation. Consequently, the rates of N_2_ fixation reported herein represent qualitative estimates of N_2_ fixation by a diverse population of diazotrophic bacterioplankton, and indicate that relatively high N_2_ fixation activity can occur in GBR waters.

Previous studies investigating diazotrophy within the water column of the GBR have either not measured N_2_ fixation rates (e.g., Hewson et al., 2007) or have measured N_2_ fixation rates by individual *Trichodesmium* trichomes using acetylene reduction (e.g., Bell et al., 1999). We propose that N_2_ fixation by the whole diazotroph community will significantly increase this estimate, and could theoretically support carbon fixation rates (assuming Redfield C:N ratios of phytoplankton) of between 0.2 and 4 μg C L^-1^ d^-1^. Although it is unlikely that all fixed N will be available to support C fixation, some autotrophic diazotrophs will directly contribute to primary production, while others may support primary production through the release of recently fixed N into the surrounding water column (Garcia et al., 2007; Lee Chen et al., 2011; Berthelot et al., 2015). For instance, the most abundant diazotroph observed in our study, the cyanobacterium *Trichodesmium erythraeum*, has been estimated to release between 50 and 90% of the N_2_ that it fixes into the surrounding environment (Glibert and Bronk, 1994; Mulholland et al., 2004), where it is potentially transferred to associated bacteria, non-diazotrophic filaments, or phytoplankton (Glibert and Bronk, 1994; Mulholland et al., 2004, 2006; Mulholland and Bernhardt, 2005).

While the fate of N fixed by heterotrophic diazotrophs remains unknown, dissolved N release from mixed, natural communities of diazotrophic bacterioplankton is in the range of 16–30% of gross whole community N_2_ fixation (Benavides et al., 2013; Bonnet et al., 2015a). Based on these numbers, we calculated potential dissolved N release, based on bulk qualitative N_2_ fixation rate measurements, in GBR waters to be between 0.4 and 20 nmol L^-1^ d^-1^. Hence diazotroph-derived dissolved N could considerably increase the potential for N_2_ fixation to support primary production within GBR waters, where ambient concentrations of DIN are considered limiting.

In the present study, ambient concentrations of DIN (NO_x_ + NH_3_) across the GBR were relatively low at <0.20 μM, but DIN concentration significantly contributed to the observed spatial heterogeneity in diazotroph community composition. Due to the reduced energy requirements associated with assimilating DIN (NO_x_ + NH_3_) compared with fixing N_2_, biological N_2_ fixation is considered to be influenced by concentrations of DIN (Karl et al., 2002; Knapp, 2012). In our study, where DIN concentrations were ≥0.11 μM we observed relatively low rates of N_2_ fixation (between 2.6 and 5.9 nmol L^-1^ d^-1^) and more diverse diazotroph communities (H′ > 2.5). Conversely, when DIN concentrations were ≤0.09 μM we observed the highest rates of N_2_ fixation (∼68 nmol L^-1^ d^-1^), associated with less diverse diazotroph communities (H′ = ∼1.5), typically dominated by *Trichodesmium* (at 30–50% of the diazotroph assemblage). In culture, cyanobacterial and proteobacterial diazotrophs have been shown to significantly decrease N_2_ fixation with increasing DIN concentrations (Knapp et al., 2012; Bentzon-Tilia et al., 2015), indicating a switch to DIN supported growth (Kustka et al., 2003; Masuda et al., 2013; Bentzon-Tilia et al., 2015), which may reduce the demand for dissolved iron (Kustka et al., 2003). However, in the environment N_2_ fixation is increasingly being found to occur outside of the classical ecological niche of low DIN waters (Fernandez et al., 2011; Knapp, 2012; Farnelid et al., 2013), and can even increase in response to simulated (mesocosm) and natural (mesoscale processes) co-additions of N with other nutrients (Dekaezemacker et al., 2013; Loscher et al., 2016). While the relationship between the availability of DIN and N_2_ fixation in the environment is more complex than perhaps previously thought, the patterns we observed suggest a significant role for DIN in structuring spatial heterogeneity in diazotroph community composition, which in turn could impact biological N_2_ fixation in GBR waters.

In addition to the influences of DIN, spatial heterogeneity in diazotrophic bacterioplankton was also significantly associated with the availability of the macro-nutrient phosphate. While ambient concentrations were generally low (<0.05 μM), sites with higher phosphate concentrations (0.024–0.049 μM) contained diazotroph communities dominated by γ-proteobacterial OTUs, while lower phosphate concentrations (0.014–0.018 μM) coincided with higher relative abundances of *Trichodesmium*. These observations may suggest differences in phosphate demand between the proteobacterial and cyanobacterial diazotrophs. Although previous phosphate enrichment experiments within GBR waters (Heron Island Lagoon) demonstrated no significant influence of phosphate on diazotroph abundance or *nifH* expression (Hewson et al., 2007), it is likely that a more complex relationship between phosphate concentration and N_2_ fixation exists within natural populations. Other sources of phosphorous, such as phosphonates (Dyhrman et al., 2006) and phosphites (Polyviou et al., 2015), may be utilized by diazotrophs *in situ*. Indeed, a recent ecosystem model that considers the availability of labile dissolved organic phosphorous (DOP) as a factor influencing diazotrophic activity, increased the estimated global N_2_ fixation budget by 30 Tg N yr^-1^ (Somes and Oschlies, 2015). Moreover, *in situ* mesocosm experiments in the tropical North Atlantic have provided direct evidence for the stimulation of N_2_ fixation after DOP addition, coinciding with a shift in diazotroph community composition (Meyer et al., 2016). Within the GBR, DOP estimates suggest concentrations similar to that of phosphate concentrations (Furnas et al., 2005), indicating that phosphate demand in GBR diazotroph communities could be met through labile DOP. Thus, the composition and abundance of GBR diazotroph assemblages are likely influenced by the availability of phosphate as well as other phosphorous sources, which, as our qualitative data indicates, may in turn significantly influence the activity of N_2_ fixation.

Overall, the findings of this study demonstrate that biological N_2_ fixation may be an important process within the pelagic realm of the GBR, where it has the potential to significantly support primary production. While we found that *Trichodesmium* dominates over spatially extensive areas of the GBR, heterotrophic N_2_-fixing bacteria may also be an important component of GBR diazotroph assemblages. Our findings indicate that diazotroph community composition is driven by the concentration of key dissolved inorganic nutrients, and in regions where DIN concentrations are low, high rates of N_2_ fixation can occur. These data highlight the need to re-evaluate N cycling dynamics within oligotrophic coral reef systems to include biological N_2_ fixation as a potentially significant source of dissolved N within the water column.

## Author Contributions

LM, MB, and JS designed the study. LM collected and processed samples, performed experiments and laboratory assays, and analyzed and interpreted the data. AM and MF provided field support, CTD data, and collected samples. RC assisted with flow cytometry and dissolved nutrient analyses. LM, MB, and JS wrote the manuscript, with input from all authors. All authors approved the manuscript.

## Conflict of Interest Statement

The authors declare that the research was conducted in the absence of any commercial or financial relationships that could be construed as a potential conflict of interest.

## References

[B1] Alongi D. M. Pfitzner J. Trott L. A. (2006). Deposition and cycling of carbon and nitrogen in carbonate mud of the lagoons of Arlington and Sudbury Reefs, Great Barrier Reef. *Coral Reefs* 25 123–143. 10.1007/s00338-005-0069-2

[B2] Bell P. R. F. Elmetri I. Uwins P. (1999). Nitrogen fixation by *Trichodesmium* spp. in the central and northern great barrier reef lagoon: relative importance of the fixed-nitrogen load. *Mar. Ecol. Prog. Ser.* 186 119–126. 10.3354/meps186119

[B3] Benavides M. Bronk D. A. Agawin N. S. R. Perez-Hernandez M. D. Hernandez-Guerra A. Aristegui J. (2013). Longitudinal variability of size-fractionated N2 fixation and DON release rates along 24.5 degrees N in the subtropical North Atlantic. *J. Geophys. Res.* 118 3406–3415. 10.1002/jgrc.20253

[B4] Bentzon-Tilia M. Severin I. Hansen L. H. (2015). Genomics and ecophysiology of heterotrophic nitrogen-fixing bacteria isolated from estuarine surface water. *MBio* 6 1–11. 10.1128/mBio.00929-15 PMC4495170 26152586

[B5] Berthelot H. Moutin T. L’Helguen S. Leblanc K. Hélias S. Grosso O. (2015). Dinitrogen fixation and dissolved organic nitrogen fueled primary production and particulate export during the VAHINE mesocosms experiment (New Caledonia lagoon). *Biogeosci. Dis.* 12 4273–4313. 10.5194/bgd-12-4273-2015

[B6] Biegala I. Raimbault P. (2008). High abundance of diazotrophic picocyanobacteria (<3 μm) in a southwest pacific coral lagoon. *Aquat. Microb. Ecol.* 51 45–53. 10.3354/ame01185

[B7] Bonnet S. Berthelot H. Turk-Kubo K. A. Fawcett S. E. Rahav E. L’Helguen S. (2015a). Dynamics of N2 fixation and fate of diazotroph-derived nitrogen during the VAHINE mesocosm experiment. *Biogeosci. Dis.* 12 19579–19626. 10.5194/bgd-12-19579-2015

[B8] Bonnet S. Rodier M. Turk-kubo K. A. Germineaud C. Menkes C. Ganachaud A. (2015b). Contrasted geographic distribution of N2 fixation rates and nifH phylotypes in the Coral and Solomon Seas (southwest Pacific) during austral winter conditions. *Glob. Biogeochem. Cycles* 29 1874–1892. 10.1002/2015GB005117

[B9] Brinkman R. Wolanski E. Deleersnijder E. McAllister F. Skirving W. (2002). Oceanic inflow from the coral sea into the great barrier reef. *Estuar. Coast. Shelf Sci.* 54 655–668. 10.1006/ecss.2001.0850

[B10] Campbell L. Carpenter E. J. Montoya J. P. Kustka A. Capone D. G. (2005). Picoplankton community structure within and outside a *Trichodesmium* bloom in the southwestern Pacfic Ocean. *Vie et Milieu* 55 185–195.

[B11] Capone D. G. Dunham S. E. Horrigan S. G. Duguay L. E. (1992). Microbial nitrogen transformations in unconsolidated coral reef sediments. *Mar. Ecol. Prog. Ser.* 80 75–88. 10.3354/meps080075

[B12] Caporaso J. Bittinger K. Bushman F. D. Desantis T. Z. Andersen G. L. Knight R. (2010a). PyNAST: a flexible tool for aligning sequences to a template alignment. *Bioinformatics* 26 266–267. 10.1093/bioinformatics/btp636 19914921 PMC2804299

[B13] Caporaso J. Kuczynski J. Stombaugh J. Bittinger K. Bushman F. Costello E. K. (2010b). QIIME allows analysis of high-throughput community sequencing data. *Nat. Methods* 7 335–336. 10.1038/nmeth0510-335 20383131 PMC3156573

[B14] Ceh J. Kilburn M. R. Cliff J. B. Raina J. B. van Keulen M. Bourne D. G. (2013). Nutrient cycling in early coral life stages: pocillopora damicornis larvae provide their algal symbiont (Symbiodinium) with nitrogen acquired from bacterial associates. *Ecol. Evol.* 3 2393–2400. 10.1002/ece3.642

[B15] Charpy L. Palinska K. A. Casareto B. Langlade M. J. Suzuki Y. Abed R. M. M. (2010). Dinitrogen-fixing cyanobacteria in microbial mats of two shallow coral reef ecosystems. *Microb. Ecol.* 59 174–186. 10.1007/s00248-009-9576-y 19705191 PMC2807599

[B16] Choukroun S. Ridd P. V. Brinkman R. McKinna L. I. W. (2010). On the surface circulation in the western coral sea and residence times in the great barrier reef. *J. Geophys. Res. Ocean.* 115 C06013 10.1029/2009JC005761

[B17] Church M. J. Jenkins B. D. Karl D. M. Zehr J. P. (2005). Vertical distributions of nitrogen-fixing phylotypes at Station ALOHA in the oligotrophic North Pacific Ocean. *Aquat. Microb. Ecol.* 38 3–14. 10.3354/ame038003

[B18] Clarke K. R. Gorley R. N. (2015). *PRIMER v7: User Manual/Tutorial*. Plymouth: PRIMER-E Ltd.

[B19] Dabundo R. Lehmann M. F. Treibergs L. Tobias C. R. Altabet M. A. Moisander P. H. (2014). The contamination of commercial 15N2 Gas Stocks with 15N–labeled nitrate and ammonium and consequences for nitrogen fixation measurements. *PLoS ONE* 9:e110335 10.1371/journal.pone.0110335 PMC4201487 25329300

[B20] Dekaezemacker J. Bonnet S. Grosso O. Moutin T. Bressac M. Capone D. G. (2013). Evidence of active dinitrogen fixation in surface waters of the eastern tropical South Pacific during El Niño and la Niña events and evaluation of its potential nutrient controls. *Glob. Biogeochem. Cycles* 27 768–779. 10.1002/gbc.20063

[B21] Devlin M. J. Brodie J. (2005). Terrestrial discharge into the great barrier reef lagoon: nutrient behavior in coastal waters. *Mar. Pollut. Bull.* 51 9–22. 10.1016/j.marpolbul.2004.10.037 15757704

[B22] Dowd S. E. Callaway T. R. Wolcott R. D. Sun Y. McKeehan T. Hagevoort R. G. (2008). Evaluation of the bacterial diversity in the feces of cattle using 16S rDNA bacterial tag-encoded FLX amplicon pyrosequencing (bTEFAP). *BMC Microbiol.* 8:125 10.1186/1471-2180-8-125 PMC2515157 18652685

[B23] Dyhrman S. T. Chappell P. D. Haley S. T. Moffett J. W. Orchard E. D. Waterbury J. B. (2006). Phosphonate utilization by the globally important marine diazotroph Trichodesmium. *Nature* 439 68–71. 10.1038/nature04203 16397497

[B24] Edgar R. C. (2010). Search and clustering orders of magnitude faster than BLAST. *Bioinformatics* 26 2460–2461. 10.1093/bioinformatics/btq461 20709691

[B25] Falter J. L. Atkinson M. J. Merrifield M. A. (2004). Mass-transfer limitation of nutrient uptake by a wave-dominated reef flat community. *Limnol. Oceanogr.* 49 1820–1831. 10.4319/lo.2004.49.5.1820

[B26] Farnelid H. Bentzon-Tilia M. Andersson A. F. Bertilsson S. Jost G. Labrenz M. (2013). Active nitrogen-fixing heterotrophic bacteria at and below the chemocline of the central Baltic Sea. *ISME J.* 7 1413–1423. 10.1038/ismej.2013.26 23446833 PMC3695292

[B27] Farnelid H. Turk-Kubo K. Del Carmen Munoz-Marin M. Zehr J. P. (2016). New insights into the ecology of the globally significant uncultured nitrogen-fixing symbiont UCYN-A. *Aquat. Microb. Ecol.* 77 128–138. 10.3354/ame01794

[B28] Fernandez C. Farías L. Ulloa O. (2011). Nitrogen fixation in denitrified marine waters. *PLoS ONE* 6:e20539 10.1371/journal.pone.0020539 PMC3110191 21687726

[B29] Fish J. A. Chai B. Wang Q. Sun Y. Brown C. T. Tiedje J. M. (2013). FunGene: the functional gene pipeline and repository. *Front. Microbiol.* 4:291 10.3389/fmicb.2013.00291 PMC3787254 24101916

[B30] Furnas M. (2003). *Catchments and Corals: Terrestrial Runoff to the Great Barrier Reef*. Townsville: Australian Institute of Marine Science.

[B31] Furnas M. Alongi D. McKinnon D. Trott L. Skuza M. (2011). Regional-scale nitrogen and phosphorus budgets for the northern (14 °S) and central (17 °S) Great Barrier Reef shelf ecosystem. *Cont. Shelf Res.* 31 1967–1990. 10.1016/j.csr.2011.09.007

[B32] Furnas M. Mitchell A. Skuza M. Brodie J. (2005). In the other 90%: phytoplankton responses to enhanced nutrient availability in the great barrier reef lagoon. *Mar. Pollut. Bull.* 51 253–265. 10.1016/j.marpolbul.2004.11.010 15757726

[B33] Furnas M. Mitchell A. W. Skuza M. (1995). *Nitrogen and Phosphorus Budgets for the Central Great Barrier Reef Shelf.* Townsville: Great Barrier Reef Marine Park Authority.

[B34] Furnas M. J. Mitchell A. W. (1996). Nutrient imputs into the central Great Barrier Reef (Australia) from subsurface intrusions of Coral Sea waters: a two-dimentional displacement model. *Cont. Shelf Res.* 16 1127–1148.

[B35] Garcia N. Raimbault P. Sandroni V. (2007). Seasonal nitrogen fixation and primary production in the Southwest Pacific: nanoplankton diazotrophy and transfer of nitrogen to picoplankton organisms. *Mar. Ecol. Prog. Ser.* 343 25–33. 10.3354/meps06882

[B36] Gasol J. M. del Giorgio P. A. (2000). Using flow cytometry for counting natural planktonic bacteria and understanding the structure of planktonic bacterial communities. *Sci. Mar.* 64 197–224. 10.3989/scimar.2000.64n2197

[B37] Glibert P. M. Bronk D. A. (1994). Release of dissolved organic nitrogen by marine diazotrophic cyanobacteria, *Trichodesmium* spp. *Appl. Environ. Microbiol.* 60 3996–4000. 16349432 10.1128/aem.60.11.3996-4000.1994 PMC201927

[B38] Großkopf T. Mohr W. Baustian T. Schunck H. Gill D. Kuypers M. M. M. (2012). Doubling of marine dinitrogen-fixation rates based on direct measurements. *Nature* 488 361–364. 10.1038/nature11338 22878720

[B39] Hearn C. Atkinson M. Falter J. (2001). A physical derivation of nutrient-uptake rates in coral reefs: effects of roughness and waves. *Coral Reefs* 20 347–356. 10.1007/s00338-001-0185-6

[B40] Heller P. Tripp H. J. Turk-Kubo K. Zehr J. P. (2014). ARBitrator: a software pipeline for on-demand retrieval of auto-curated nifH sequences from GenBank. *Bioinformatics* 30 1–8. 10.1093/bioinformatics/btu417 24990605

[B41] Hewson I. Moisander P. H. Morrison A. E. Zehr J. P. (2007). Diazotrophic bacterioplankton in a coral reef lagoon: phylogeny, diel nitrogenase expression and response to phosphate enrichment. *ISME J.* 1 78–91. 10.1038/ismej.2007.5 18043616

[B42] Holmes R. M. Aminot A. Kerouel R. Hooker B. A. Peterson B. J. (1999). A simple and precise method for measuring ammonium in marine and freshwater ecosystems. *Can. J. Fish. Aquat. Sci.* 56 1801–1808. 10.1139/cjfas-56-10-1801

[B43] Karl D. Michaels A. Bergman B. Capone D. Carpenter E. Letelier R. (2002). Dinitrogen fixation in the world’s oceans. *Biogeochemistry* 5 47–98. 10.1023/A:1015798105851

[B44] Klawonn I. Lavik G. Böning P. Marchant H. K. Dekaezemacker J. Mohr W. (2015). Simple approach for the preparation of 15N2-enriched water for nitrogen fixation assessments: evaluation, application and recommendations. *Front. Microbiol.* 6:769 10.3389/fmicb.2015.00769 PMC4523818 26300853

[B45] Knapp A. Dekaezemacker J. Bonnet S. Sohm J. Capone D. (2012). Sensitivity of *Trichodesmium erythraeum* and *Crocosphaera watsonii* abundance and N2 fixation rates to varying NO_3_^-^ and PO_4_^3-^ concentrations in batch cultures. *Aquat. Microb. Ecol.* 66 223–236. 10.3354/ame01577

[B46] Knapp A. N. (2012). The sensitivity of marine N2 fixation to dissolved inorganic nitrogen. *Front. Microbiol.* 3:374 10.3389/fmicb.2012.00374 PMC3476826 23091472

[B47] Kumar S. Stecher G. Tamura K. (2016). MEGA7: molecular evolutionary genetics analysis version 7.0 for bigger datasets. *Mol. Biol. Evol.* 33:msw054 10.1093/molbev/msw054 PMC8210823 27004904

[B48] Kustka A. B. Sañudo-Wilhelmy S. A. Carpenter E. J. Capone D. Burns J. Sunda W. G. (2003). Iron requirements for dinitrogen- and ammonium-supported growth in cultures of Trichodesmium (IMS 101): comparison with nitrogen fixation rates and iron:carbon ratios of field populations. *Limnol. Oceanogr.* 48 1869–1884. 10.4319/lo.2003.48.5.1869

[B49] Langlois R. Großkopf T. Mills M. Takeda S. LaRoche J. (2015). Widespread distribution and expression of Gamma A (UMB), an uncultured, diazotrophic, γ-Proteobacterial nifH Phylotype. *PLoS ONE* 10:e0128912 10.1371/journal.pone.0128912 PMC4477881 26103055

[B50] Larkum A. W. D. Kennedy I. R. Muller W. J. (1988). Nitrogen fixation on a coral reef. *Mar. Biol.* 98 143–155. 10.2216/i0031-8884-14-2-87.1

[B51] Lee Chen Y. Tuo S. Chen H. (2011). Co-occurrence and transfer of fixed nitrogen from *Trichodesmium* spp. to diatoms in the low-latitude Kuroshio Current in the NW Pacific. *Mar. Ecol. Prog. Ser.* 421 25–38. 10.3354/meps08908

[B52] Lema K. A. Willis B. L. Bourne D. G. (2012). Corals form characteristic associations with symbiotic nitrogen-fixing bacteria. *Appl. Environ. Microbiol.* 78 3136–3144. 10.1128/AEM.07800-11 22344646 PMC3346485

[B53] Lema K. A. Willis B. L. Bourne D. G. (2014). Amplicon pyrosequencing reveals spatial and temporal consistency in diazotroph assemblages of the *Acropora millepora* microbiome. *Environ. Microbiol.* 16 3345–3359. 10.1111/1462-2920.12366 24373029

[B54] Lesser M. P. Falcón L. I. Rodríguez-Román A. Enríquez S. Hoegh-Guldberg O. Iglesias-Prieto R. (2007). Nitrogen fixation by symbiotic cyanobacteria provides a source of nitrogen for the scleractinian coral *Montastraea cavernosa*. *Mar. Ecol. Prog. Ser.* 346 143–152. 10.3354/meps07008

[B55] Lesser M. P. Mazel C. H. Gorbunov M. Y. Falkowski P. G. (2004). Discovery of symbiotic nitrogen-fixing cyanobacteria in corals. *Science* 305 997–1000. 15310901 10.1126/science.1099128

[B56] Loscher C. R. Bourbonnais A. Dekaezemacker J. Charoenpong C. N. Altabet M. A. Bange H. W. (2016). N2 fixation in eddies of the eastern tropical South Pacific Ocean. *Biogeosciences* 13 2889–2899. 10.5194/bg-13-2889-2016

[B57] Luo Y. W. Doney S. C. Anderson L. A. Benavides M. Berman-Frank I. Bode A. (2012). Database of diazotrophs in global ocean: abundance, biomass and nitrogen fixation rates. *Earth Syst. Sci. Data* 4 47–73. 10.5194/essd-4-47-2012

[B58] Marie D. Partensky F. Jacquet S. Vaulot D. (1997). Enumeration and cell cycle analysis of natural populations of marine picoplankton by flow cytometry using the nucleic acid stain SYBR Green I. *Appl. Environ. Microbiol.* 63 186–193. 10.1111/j.1365-294X.2009.04480.x 16535483 PMC1389098

[B59] Masuda T. Furuya K. Kodama T. Takeda S. Harrison P. J. (2013). Ammonium uptake and dinitrogen fixation by the unicellular nanocyanobacterium *Crocosphaera watsonii* in nitrogen-limited continuous cultures. *Limnol. Oceanogr.* 58 2029–2036. 10.4319/lo.2013.58.6.2029

[B60] McKinna L. Furnas M. Ridd P. (2011). A simple, binary classification algorithm for the detection of *Trichodesmium* spp. within the Great Barrier Reef using MODIS imagery. *Limnol. Oceanogr. Methods* 9 50–66. 10.4319/lom.2011.9.50

[B61] Messer L. F. Doubell M. Jeffries T. C. Brown M. V. Seymour J. R. (2015a). Prokaryotic and diazotrophic population dynamics within a large oligotrophic inverse estuary. *Aquat. Microb. Ecol.* 74 1–15. 10.3354/ame01726

[B62] Messer L. F. Mahaffey C. Robinson C. M. Jeffries T. C. Baker K. G. Isaksson J. B. (2015b). High levels of heterogeneity in diazotroph diversity and activity within a putative hotspot for marine nitrogen fixation. *ISME J.* 10 1499–1513. 10.1038/ismej.2015.205 26613341 PMC5029180

[B63] Meyer J. Loscher C. R. Neulinger S. C. Reichel A. F. Loginova A. Borchard C. (2016). Changing nutrient stoichiometry affects phytoplankton production, DOP accumulation and dinitrogen fixation – a mesocosm experiment in the eastern tropical North Atlantic. *Biogeosciences* 13 781–794. 10.5194/bg-13-781-2016

[B64] Mohr W. Großkopf T. Wallace D. W. R. LaRoche J. (2010). Methodological underestimation of oceanic nitrogen fixation rates. *PLoS ONE* 5:e12583 10.1371/journal.pone.0012583 PMC2933240 20838446

[B65] Moisander P. H. Beinart R. A. Hewson I. White A. E. Johnson K. S. Carlson C. A. (2010). Unicellular cyanobacterial distributions broaden the oceanic N2 fixation domain. *Science* 327 1512–1514. 10.1126/science.1185468 20185682

[B66] Moisander P. H. Beinart R. A. Voss M. Zehr J. P. (2008). Diversity and abundance of diazotrophic microorganisms in the South China Sea during intermonsoon. *ISME J.* 2 954–967. 10.1038/ismej.2008.84 18528417

[B67] Moisander P. H. Serros T. Paerl R. W. Beinart R. A. Zehr J. P. (2014). Gammaproteobacterial diazotrophs and nifH gene expression in surface waters of the South Pacific Ocean. *ISME J.* 8 1962–1973. 10.1038/ismej.2014.49 24722632 PMC4184014

[B68] Montoya J. P. Voss M. Kahler P. Capone D. G. (1996). A simple, high-precision, high-sensitivity tracer assay for N2 fixation. *Appl. Environ. Microbiol.* 62 986–993. 16535283 10.1128/aem.62.3.986-993.1996 PMC1388808

[B69] Mulholland M. R. Bernhardt P. W. (2005). The effect of growth rate, phosphorus concentration, and temperature on N2 fixation, carbon fixation, and nitrogen release in continuous cultures of *Trichodesmium* IMS101. *Limnol. Oceanogr.* 50 839–849. 10.4319/lo.2005.50.3.0839

[B70] Mulholland M. R. Bernhardt P. W. Heil C. A. Bronk D. A. Neil J. M. O. (2006). Nitrogen fixation and release of fixed nitrogen by *Trichodesmium* spp. in the Gulf of Mexico. *Limnol. Oceanogr.* 51 1762–1776. 10.4319/lo.2006.51.4.1762

[B71] Mulholland M. R. Mulholland M. R. Bronk D. A. Bronk D. A. Capone D. G. Capone D. G. (2004). Dinitrogen fixation and release of ammonium and dissolved organic nitrogen by *Trichodesmium* IMS101. *Aquat. Microb. Ecol.* 37 85–94.

[B72] Olson N. D. Lesser M. P. (2013). Diazotrophic diversity in the Caribbean coral, *Montastraea cavernosa*. *Arch. Microbiol.* 195 853–859. 10.1007/s00203-013-0937-z 24217873

[B73] Polyviou D. Hitchcock A. Baylay A. J. Moore C. M. Bibby T. S. (2015). Phosphite utilisation by the globally important marine diazotroph *Trichodesmium*. *Environ. Microbiol. Rep.* 7 824–830. 10.1111/1758-2229.12308 26081517

[B74] Santos H. F. Carmo F. L. Duarte G. Dini-Andreote F. Castro C. B. Rosado A. S. (2014). Climate change affects key nitrogen-fixing bacterial populations on coral reefs. *ISME J.* 8 2272–2279. 10.1038/ismej.2014.70 24830827 PMC4992079

[B75] Schaffelke B. Carleton J. Skuza M. Zagorskis I. Furnas M. J. (2012). Water quality in the inshore great barrier reef lagoon: implications for long-term monitoring and management. *Mar. Pollut. Bull.* 65 249–260. 10.1016/j.marpolbul.2011.10.031 22142496

[B76] Seymour J. R. Seuront L. Mitchell J. G. (2007). Microscale gradients of planktonic microbial communities above the sediment surface in a mangrove estuary. *Estuar. Coast. Shelf Sci.* 73 651–666. 10.1016/j.ecss.2007.03.004

[B77] Somes C. J. Oschlies A. (2015). On the influence of “non-Redfield” dissolved organic nutrient dynamics on the spatial distribution of N_2_ fixation and the size of the marine fixed nitrogen inventory. *Global Biogeochem. Cycles* 29 973–993. 10.1002/2014GB005050

[B78] Tamura K. Nei M. (1993). Estimation of the number of nucleotide substitutions in the control region of mitochondrial DNA in humans and chimpanzees. *Mol. Biol. Evol.* 10 512–526. 10.1093/molbev/msl149 8336541

[B79] Turk-Kubo K. A. Achilles K. M. Serros T. R. C. Ochiai M. Montoya J. P. Zehr J. P. (2012). Nitrogenase (nifH) gene expression in diazotrophic cyanobacteria in the Tropical North Atlantic in response to nutrient amendments. *Front. Microbiol.* 3:368 10.3389/fmicb.2012.00386 PMC3487379 23130017

[B80] Turk-Kubo K. A. Frank I. E. Hogan M. E. Desnues A. Bonnet S. Zehr J. P. (2015). Diazotroph community succession during the VAHINE mesocosm experiment (New Caledonia lagoon). *Biogeosciences* 12 7435–7452. 10.5194/bg-12-7435-2015

[B81] Waterhouse J. Brodie J. Lewis S. Mitchell A. (2012). Quantifying the sources of pollutants in the great barrier reef catchments and the relative risk to reef ecosystems. *Mar. Pollut. Bull.* 65 394–406. 10.1016/j.marpolbul.2011.09.031 22070980

[B82] Webb K. L. Dupaul W. D. Wiebe W. Sottile W. Johannes R. E. (1975). Enewetak(Eniwetok) atoll: aspects of the nitrogen cycle on a coral reef. *Limnol. Oceanogr.* 20 198–210. 10.4319/lo.1975.20.2.0198

[B83] Wiebe W. J. Johannes R. E. Webb K. L. (1975). Nitrogen fixation in a coral reef community. *Science* 188 257–259. 10.1126/science.188.4185.257 17800402

[B84] Wilson S. T. Bottjer D. Church M. J. Karl D. M. (2012). comparative assessment of nitrogen fixation methodologies, conducted in the oligotrophic north pacific ocean. *Appl. Environ. Microbiol.* 78 6516–6523. 10.1128/AEM.01146-12 22773638 PMC3426697

[B85] Woebken D. Burow L. C. Behnam F. Mayali X. Schintlmeister A. Fleming E. D. (2014). Revisiting N2 fixation in guerrero negro intertidal microbial mats with a functional single-cell approach. *ISME J.* 9144 1–12. 10.1038/ismej.2014.144 PMC4303640 25303712

[B86] Zehr J. P. Jenkins B. D. Short S. M. Steward G. F. (2003). Nitrogenase gene diversity and microbial community structure: a cross-system comparison. *Environ. Microbiol.* 5 539–554. 12823187 10.1046/j.1462-2920.2003.00451.x

[B87] Zehr J. P. McReynolds L. A. (1989). Use of degenerate oligonucleotides for amplification of the nifH gene from the marine cyanobacterium *Trichodesmium thiebautii*. *Appl. Environ. Microbiol.* 55 2522–2526. 2513774 10.1128/aem.55.10.2522-2526.1989 PMC203115

[B88] Zehr J. P. Shilova I. N. Farnelid H. M. del Carmen Muñoz-Marín M. Turk-Kubo K. A. (2016). Unusual marine unicellular symbiosis with the nitrogen-fixing cyanobacterium UCYN-A. *Nat. Microbiol.* 2:16214 10.1038/nmicrobiol.2016.214 27996008

[B89] Zehr J. P. Turner P. J. (2001). Nitrogen fixation: nitrogenase genes and gene expression. *Methods Microbiol.* 30 271–285.

[B90] Zhang Y. Yang Q. Ling J. Van Nostrand J. D. Shi Z. Zhou J. (2016). The shifts of diazotrophic communities in spring and summer associated with coral *Galaxea astreata, Pavona decussata*, and *Porites lutea*. *Front. Microbiol.* 7:1870 10.3389/fmicb.2016.01870 PMC5118425 27920768

