# Supplementary material for: Diversity and Activity of Diazotrophs in Great Barrier Reef Surface Waters
Source: Front Microbiol. 2017 Jun 7;8:967. doi: 10.3389/fmicb.2017.00967 (PMC5461343; doi:10.3389/fmicb.2017.00967)
Supplement: Supplementary file 4 [file Image_1.PDF]

Supplementary Material for:

Diversity and activity of diazotrophs in Great Barrier Reef surface waters

Lauren F. Messer[1,2*], Mark V. Brown[2], Miles J. Furnas[3], Richard Carney[1], A. David McKinnon[3], and Justin R. Seymour[1]

1. Climate Change Cluster, University of Technology Sydney, Sydney, New South Wales, Australia
2. School of Biotechnology and Biomolecular Sciences, University of New South Wales, Sydney, New South Wales, Australia
3. Australian Institute of Marine Science, Townsville, Queensland, Australia

* Correspondence:

Lauren Messer

laurenfrances.messer@student.uts.edu.au.

Table and Figure Legends

Supplementary Table 1. Sequencing and quality control statistics.

Supplementary Table 2. Results from the distance-based linear modelling. Significant predictor variables are shown in bold.

Supplementary Table 3. Sigma Aldrich gas potential contamination calculation.

Supplementary Figure 1. Temperature and salinity plot of CTD profiles from the ten sampling locations, with isopycnals overlaid. Sampling depths (5m) are indicated with a black arrow.

Supplementary Figure 2. Maximum likelihood phylogenetic tree of the 92 *nifH* OTUs recovered in this study and representative cultured and environmental *nifH* sequences. Bootstraps with over 50% support are shown for 1000 bootstraps.

Supplementary Figure 3. Redundancy Analysis plot with significant variables identified by distance-based redundancy analysis overlaid as vectors.

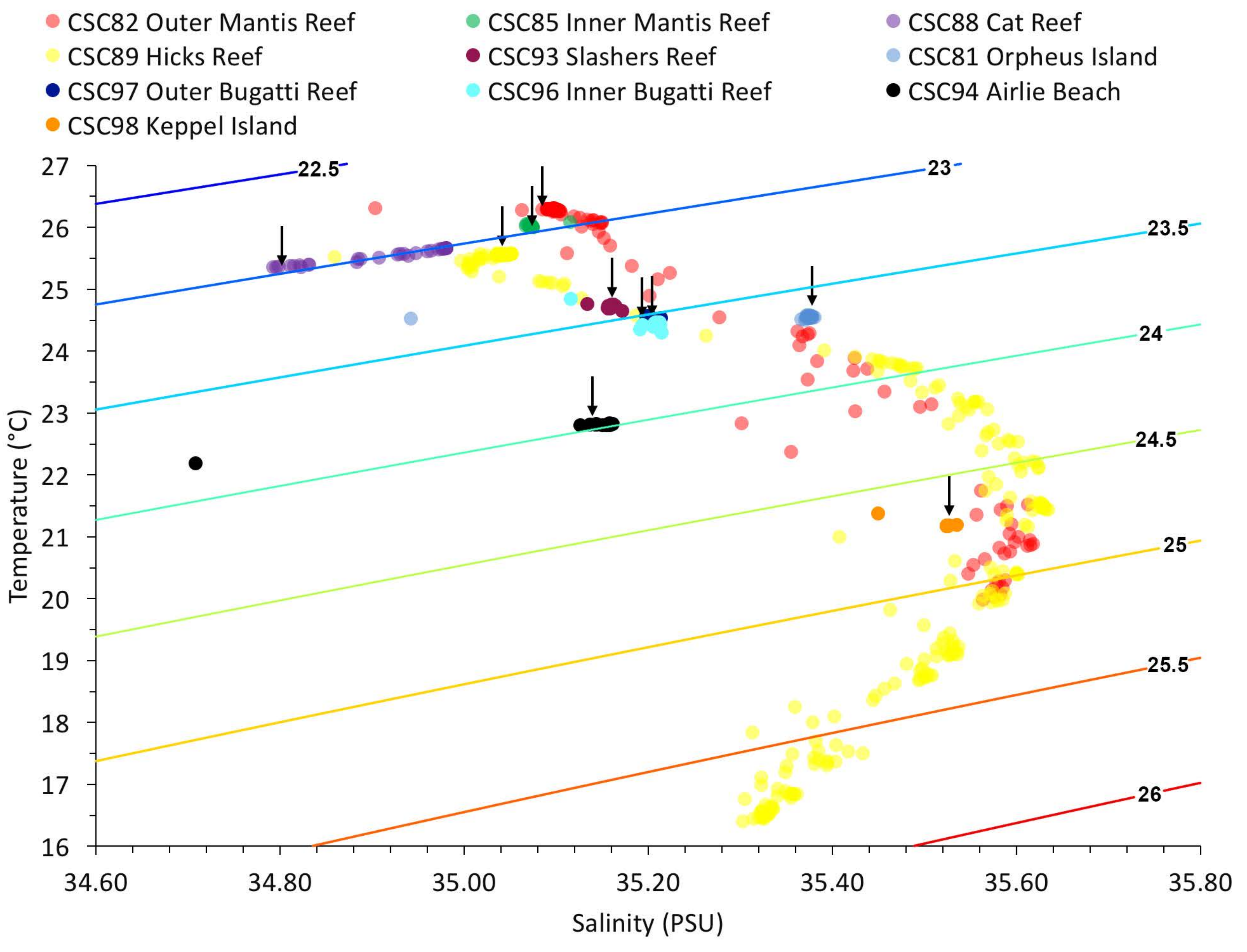

CSC82 Outer Mantis Reef
CSC85 Inner Mantis Reef
CSC88 Cat Reef
CSC89 Hicks Reef
CSC93 Slashers Reef
CSC81 Orpheus Island
CSC97 Outer Bugatti Reef
CSC96 Inner Bugatti Reef
CSC94 Airlie Beach
CSC98 Keppel Island
22.5
23
23.5
24
24.5
25
25.5
26
Temperature (°C)
Salinity (PSU)
27
26
25
24
23
22
21
20
19
18
17
16
34.60
34.80
35.00
35.20
35.40
35.60
35.80

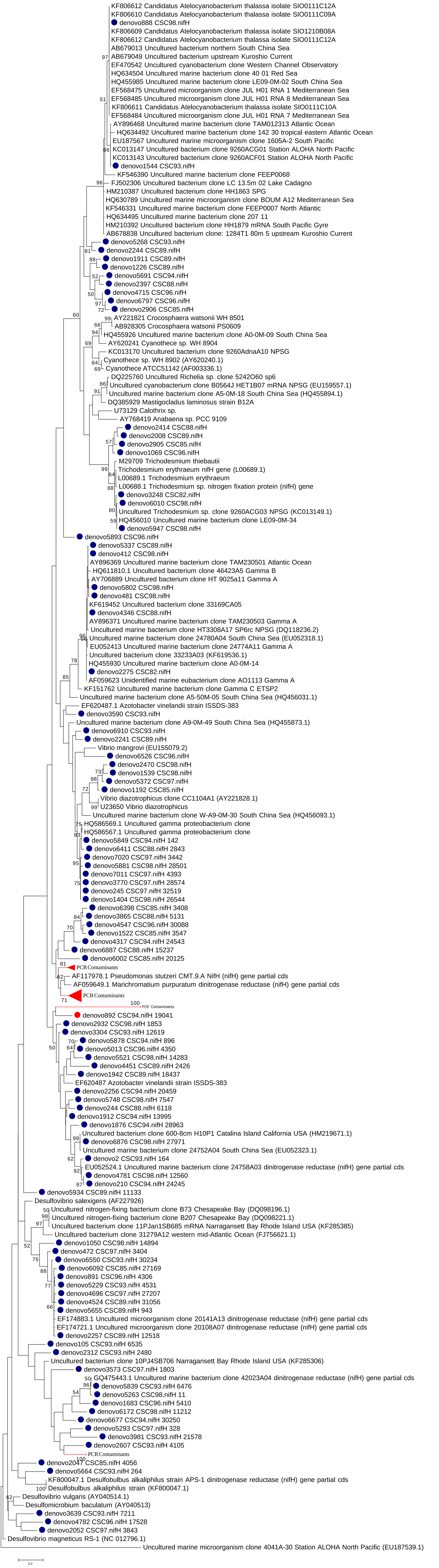
KF806612 Candidatus Atelocyanobacterium thalassa isolate SIO0111C12A
KF806610 Candidatus Atelocyanobacterium thalassa isolate SIO0111C09A
denovo888 CSC98.nifH
KF806609 Candidatus Atelocyanobacterium thalassa isolate SIO1210B08A
KF806612 Candidatus Atelocyanobacterium thalassa isolate SIO0111C12A
AB679013 Uncultured bacterium northern South China Sea
AB679049 Uncultured bacterium upstream Kuroshio Current
EF470542 Uncultured cyanobacterium clone Western Channel Observatory
HQ634504 Uncultured marine bacterium clone 40 01 Red Sea
HQ455985 Uncultured marine bacterium clone LE09-0M-02 South China Sea
EF568475 Uncultured microorganism clone JUL H01 RNA 1 Mediterranean Sea
EF568485 Uncultured microorganism clone JUL H01 RNA 8 Mediterranean Sea
KF806611 Candidatus Atelocyanobacterium thalassa isolate SIO0111C10A
EF568484 Uncultured microorganism clone JUL H01 RNA 7 Mediterranean Sea
AY896468 Uncultured marine bacterium clone TAM012313 Atlantic Ocean
HQ634492 Uncultured marine bacterium clone 142 30 tropical eastern Atlantic Ocean
EU187567 Uncultured marine microorganism clone 1605A-2 South Pacific
KC013147 Uncultured marine bacterium clone 9260ACG01 Station ALOHA North Pacific
KC013143 Uncultured marine bacterium clone 9260ACF01 Station ALOHA North Pacific
denovo1544 CSC93.nifH
KF546390 Uncultured marine bacterium clone FEEP0068
FJ502306 Uncultured bacterium clone LC 13.5m 02 Lake Cadagno
HM210387 Uncultured bacterium clone HH1863 SPG
HQ630789 Uncultured marine microorganism clone BOUM A12 Mediterranean Sea
KF546331 Uncultured marine bacterium clone FEEP0007 North Atlantic
HQ634495 Uncultured marine bacterium clone 207 11
HM210392 Uncultured bacterium clone HH1879 mRNA South Pacific Gyre
AB678838 Uncultured bacterium clone: 1284T1 80m 5 upstream Kuroshio Current
denovo5268 CSC93.nifH
denovo2244 CSC89.nifH
denovo1911 CSC89.nifH
denovo1226 CSC89.nifH
denovo5691 CSC94.nifH
denovo2397 CSC88.nifH
denovo4715 CSC96.nifH
denovo6797 CSC96.nifH
denovo2906 CSC85.nifH
AY221821 Crocosphaera watsonii WH 8501
AB928305 Crocosphaera watsonii PS0609
HQ455926 Uncultured marine bacterium clone A0-0M-09 South China Sea
AY620241 Cyanothece sp. WH 8904
KC013170 Uncultured cyanobacterium clone 9260AdnaA10 NPSG
Cyanothece sp. WH 8902 (AY620240.1)
Cyanothece ATCC51142 (AF003336.1)
DQ225760 Uncultured Richelia sp. clone 5242O60 sp6
Uncultured cyanobacterium clone B0564J HET1B07 mRNA NPSG (EU159557.1)
Uncultured marine bacterium clone A5-0M-18 South China Sea (HQ455894.1)
DQ385929 Mastigocladus laminosus strain B12A
U73129 Calothrix sp.
AY768419 Anabaena sp. PCC 9109
denovo2414 CSC88.nifH
denovo2008 CSC89.nifH
denovo2905 CSC85.nifH
denovo1069 CSC96.nifH
M29709 Trichodesmium thiebautii
Trichodesmium erythraeum nifH gene (L00689.1)
L00689.1 Trichodesmium erythraeum
L00688.1 Trichodesmium sp. nitrogen fixation protein (nifH) gene
denovo3248 CSC82.nifH
denovo6010 CSC98.nifH
Uncultured Trichodesmium sp. clone 9260ACG03 NPSG (KC013149.1)
HQ456010 Uncultured marine bacterium clone LE09-0M-34
denovo5947 CSC98.nifH
denovo5893 CSC96.nifH
denovo5337 CSC89.nifH
denovo412 CSC98.nifH
AY896369 Uncultured marine bacterium clone TAM230501 Atlantic Ocean
HQ611810.1 Uncultured bacterium clone 46423A5 Gamma B
AY706889 Uncultured bacterium clone HT 9025a11 Gamma A
denovo5802 CSC98.nifH
denovo481 CSC98.nifH
KF619452 Uncultured bacterium clone 33169CA05
denovo4346 CSC88.nifH
AY896371 Uncultured marine bacterium clone TAM230503 Gamma A
Uncultured marine bacterium clone HT3308A17 SP6rc NPSG (DQ118236.2)
Uncultured marine bacterium clone 24780A04 South China Sea (EU052318.1)
EU052413 Uncultured marine bacterium clone 24774A11 Gamma A
Uncultured bacterium clone 33233A03 (KF619536.1)
HQ455930 Uncultured marine bacterium clone A0-0M-14
denovo2275 CSC82.nifH
AF059623 Unidentified marine eubacterium clone AO1113 Gamma A
KF151762 Uncultured marine bacterium clone Gamma C ETSP2
Uncultured marine bacterium clone A5-50M-05 South China Sea (HQ456031.1)
EF620487.1 Azotobacter vinelandii strain ISSDS-383
denovo3590 CSC93.nifH
Uncultured marine bacterium clone A9-0M-49 South China Sea (HQ455873.1)
denovo6910 CSC93.nifH
denovo2241 CSC89.nifH
Vibrio mangrovi (EU155079.2)
denovo6526 CSC96.nifH
denovo2470 CSC98.nifH
denovo1539 CSC98.nifH
denovo5372 CSC97.nifH
denovo1192 CSC85.nifH
Vibrio diazotrophicus clone CC1104A1 (AY221828.1)
U23650 Vibrio diazotrophicus
Uncultured marine bacterium clone W-A9-0M-30 South China Sea (HQ456093.1)
HQ586569.1 Uncultured gamma proteobacterium clone
HQ586567.1 Uncultured gamma proteobacterium clone
denovo5849 CSC94.nifH 142
denovo6411 CSC88.nifH 2843
denovo7020 CSC97.nifH 3442
denovo5881 CSC98.nifH 28501
denovo7011 CSC97.nifH 4393
denovo3770 CSC97.nifH 28574
denovo245 CSC97.nifH 32519
denovo1404 CSC98.nifH 26544
denovo6398 CSC85.nifH 3408
denovo3865 CSC88.nifH 5131
denovo4547 CSC96.nifH 30088
denovo1522 CSC85.nifH 3547
denovo4317 CSC94.nifH 24543
denovo6887 CSC88.nifH 15237
denovo6002 CSC85.nifH 20125
PCR Contaminants
AF117978.1 Pseudomonas stutzeri CMT.9.A NifH (nifH) gene partial cds
AF059649.1 Marichromatium purpuratum dinitrogenase reductase (nifH) gene partial cds
PCR Contaminants
PCR Contaminants
denovo892 CSC94.nifH 19041
denovo2932 CSC98.nifH 1853
denovo3304 CSC93.nifH 12619
denovo5878 CSC94.nifH 896
denovo5013 CSC96.nifH 4350
denovo5521 CSC98.nifH 14283
denovo4451 CSC89.nifH 2426
denovo1942 CSC89.nifH 18437
EF620487 Azotobacter vinelandii strain ISSDS-383
denovo2256 CSC94.nifH 20459
denovo5748 CSC98.nifH 7547
denovo244 CSC88.nifH 6118
denovo1912 CSC94.nifH 13995
denovo1876 CSC94.nifH 28963
Uncultured bacterium clone 600-8cm H10P1 Catalina Island California USA (HM219671.1)
denovo6876 CSC98.nifH 27971
Uncultured marine bacterium clone 24752A04 South China Sea (EU052323.1)
denovo2 CSC93.nifH 164
EU052524.1 Uncultured marine bacterium clone 24758A03 dinitrogenase reductase (nifH) gene partial cds
denovo4781 CSC98.nifH 12560
denovo210 CSC94.nifH 24245
denovo5934 CSC89.nifH 11133
Desulfovibrio salexigens (AF227926)
Uncultured nitrogen-fixing bacterium clone B73 Chesapeake Bay (DQ098196.1)
Uncultured nitrogen-fixing bacterium clone B207 Chesapeake Bay (DQ098221.1)
Uncultured bacterium clone 11PJan1SB685 mRNA Narragansett Bay Rhode Island USA (KF285385)
Uncultured bacterium clone 31279A12 western mid-Atlantic Ocean (FJ756621.1)
denovo1050 CSC98.nifH 14894
denovo472 CSC97.nifH 3404
denovo6550 CSC93.nifH 30234
denovo6092 CSC85.nifH 27169
denovo891 CSC96.nifH 4306
denovo5229 CSC93.nifH 4531
denovo4696 CSC97.nifH 27207
denovo4524 CSC89.nifH 31056
denovo5655 CSC89.nifH 943
EF174883.1 Uncultured microorganism clone 20141A13 dinitrogenase reductase (nifH) gene partial cds
EF174721.1 Uncultured microorganism clone 20108A07 dinitrogenase reductase (nifH) gene partial cds
denovo2257 CSC89.nifH 12518
denovo105 CSC93.nifH 6535
denovo2312 CSC93.nifH 2480
Uncultured bacterium clone 10PJ4SB706 Narragansett Bay Rhode Island USA (KF285306)
denovo3573 CSC97.nifH 1803
GQ475443.1 Uncultured marine bacterium clone 42023A04 dinitrogenase reductase (nifH) gene partial cds
denovo5839 CSC93.nifH 6476
denovo5263 CSC98.nifH 11
denovo1683 CSC96.nifH 5410
denovo6172 CSC98.nifH 11212
denovo6677 CSC94.nifH 30250
denovo5293 CSC97.nifH 328
denovo3981 CSC93.nifH 21578
denovo2607 CSC93.nifH 4105
PCR Contaminants
denovo2047 CSC85.nifH 4056
denovo5664 CSC93.nifH 264
KF800047.1 Desulfobulbus alkaliphilus strain APS-1 dinitrogenase reductase (nifH) gene partial cds
Desulfobulbus alkaliphilus strain (KF800047.1)
Desulfovibrio vulgaris (AY040514.1)
Desulfomicrobium baculatum (AY040513)
denovo3639 CSC93.nifH 7211
denovo4782 CSC96.nifH 17528
denovo2052 CSC97.nifH 3843
Desulfovibrio magneticus RS-1 (NC 012796.1)
Uncultured marine microorganism clone 4041A-30 Station ALOHA North Pacific (EU187539.1)
0.2

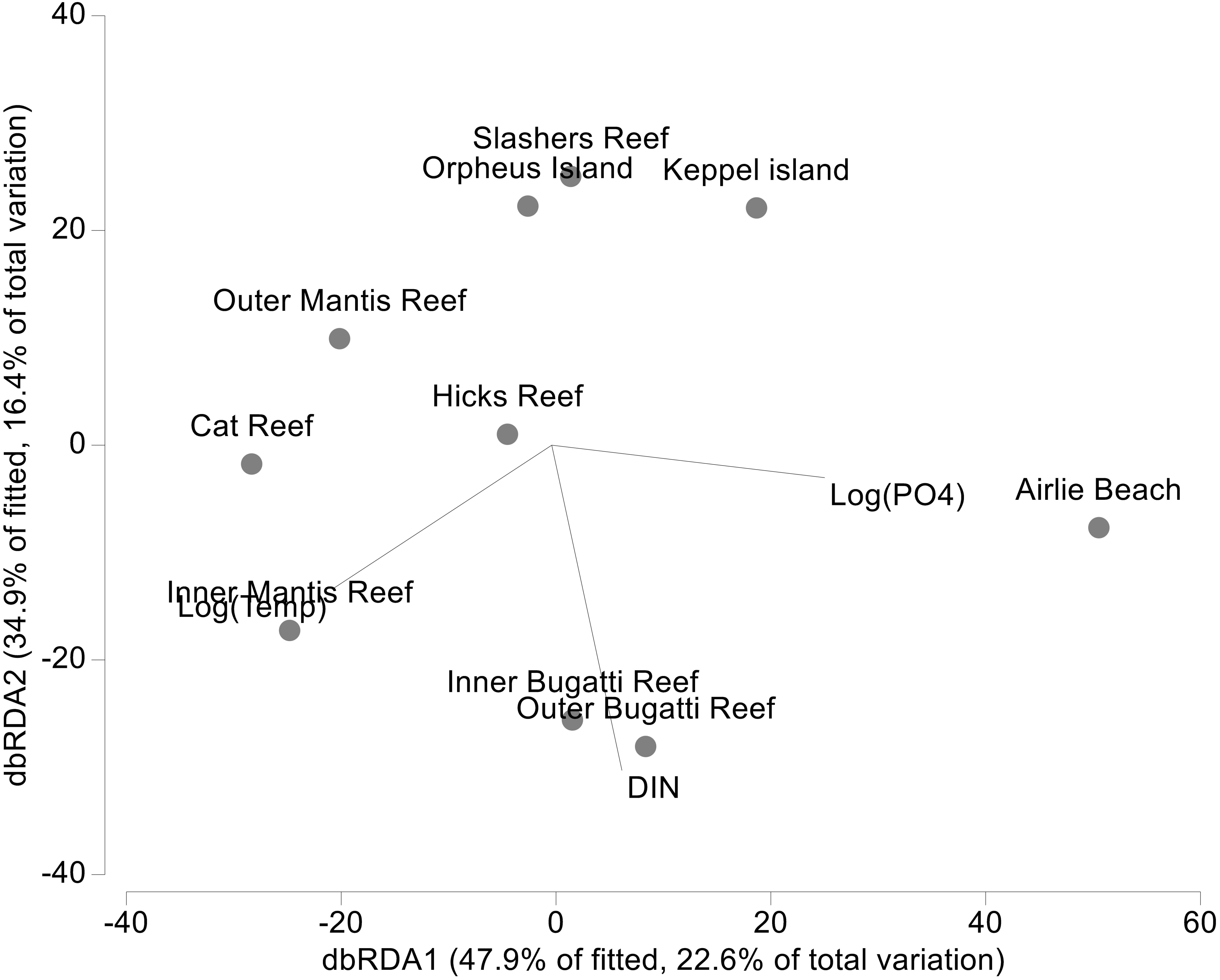

Slashers Reef
Orpheus Island
Keppel island
Outer Mantis Reef
Hicks Reef
Cat Reef
Log(PO4)
Airlie Beach
Inner Mantis Reef
Log(Temp)
Inner Bugatti Reef
Outer Bugatti Reef
DIN
40
20
0
-20
-40
-40
-20
0
20
40
60
dbRDA2 (34.9% of fitted, 16.4% of total variation)
dbRDA1 (47.9% of fitted, 22.6% of total variation)
